# Effects of high light and elevated temperature on the structure of photosystem II isolated from *Arabidopsis thaliana*

**DOI:** 10.1007/s11120-026-01238-w

**Published:** 2026-10-08

**Authors:** Jack Forsman, André T. Graça, Michael Hall, Wolfgang P. Schröder, Johannes Messinger

**Affiliations:** 1 Department of Chemistry, Umeå University, Umeå, SE 90187 Sweden; 2 Molecular Biomimetics, Department of Chemistry-Ångström, Uppsala University, Uppsala, SE 75120 Sweden; 3 European Molecular Biology Laboratory, EMBL Grenoble, Grenoble, 38042 France; 4 Umeå Plant Science Center, Department of Plant Physiology, Umeå University, Umeå, SE 90187 Sweden

**Keywords:** Photosynthesis, Photosystem II, Structure, Cryo-EM, High light, High temperature

## Abstract

Photosystem II (PS II) is a large membrane-bound pigment-protein complex responsible for extracting electrons and protons from water during photosynthesis and releasing molecular oxygen into the atmosphere. While light is required to drive the photochemical reactions, high light and high temperature can be damaging to PS II. To gain insight into potential structural consequences of such conditions, we performed an exploratory study where isolated PS II complexes (C2S2) from *Arabidopsis thaliana* were exposed to high light and elevated temperature and employed cryo-electron microscopy for structural determination. This resulted in a ‘high light’ structure (2.74 Å global resolution) which is compared to the published 2.44 Å structure of intact Arabidopsis-PSII vitrified under dim light conditions (control). The high light structure is missing several of the subunits present in the control, including the strongly bound light-harvesting antenna complexes as well as several small transmembrane and extrinsic subunits. Beyond the loss of subunits, the clearest evidence of specific photodamage was found on the acceptor side of the D1 and D2 subunits near the non-heme iron, including the loss of the bicarbonate ligand of the non-heme iron. Finally, we were able to observe that high light/elevated temperature conditions result in the movement of the D1:His252 residue, potentially forming a serine protease triad that may trigger auto-proteolysis of the D1 subunit.

**Supplementary Information:**

The online version contains supplementary material available at https://doi.org/10.1007/s11120-026-01238-w.

## Introduction

**Fig. 1:**
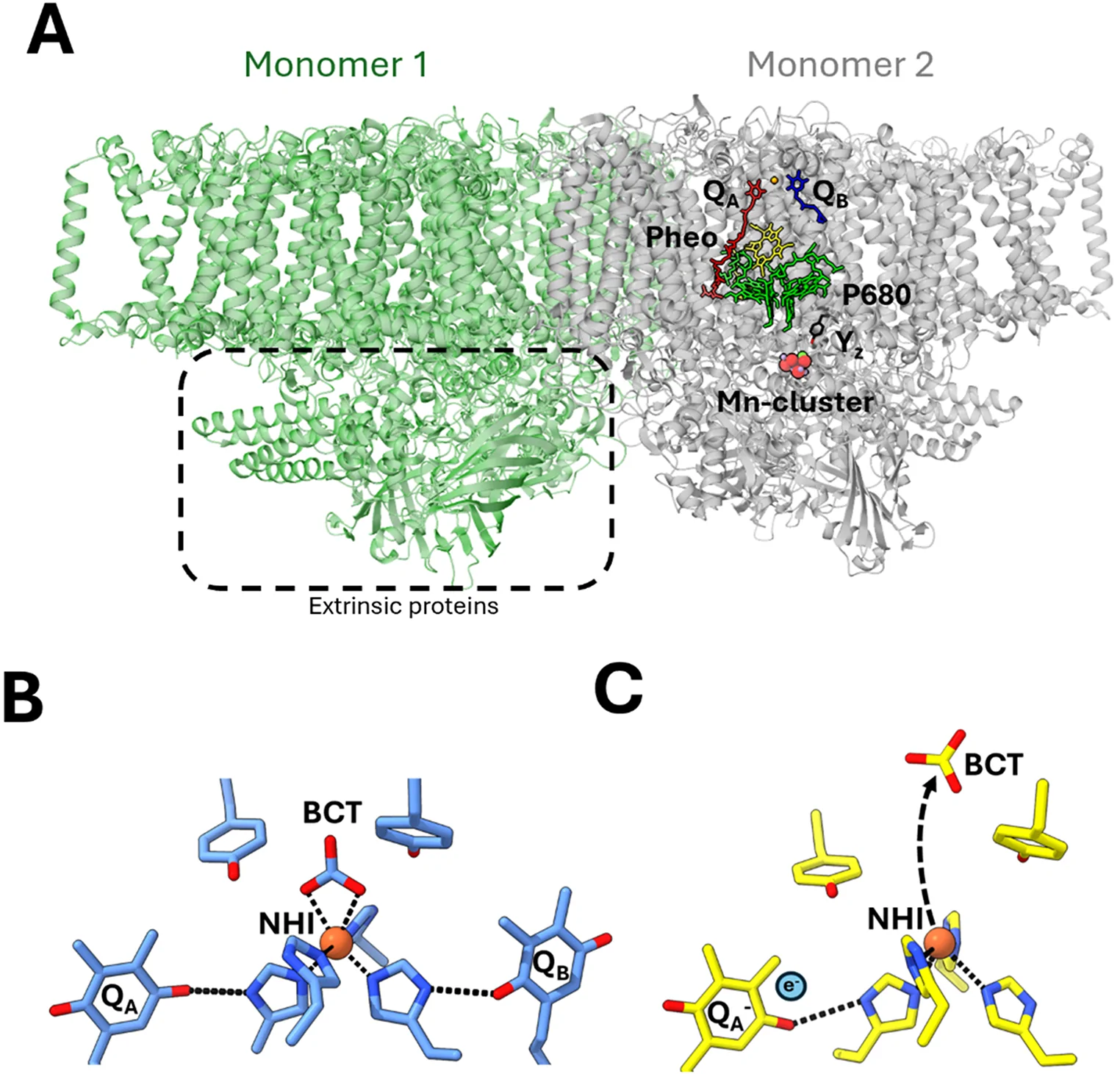
**(A)** Photosystem II dimer from *Arabidopsis thaliana* handled in dim light (control) conditions (side view) (pdb:9I4T). Monomer 1 is shown in green, monomer 2 is shown in grey. Extrinsic proteins are highlighted in Monomer 1 with a dashed square. Selected cofactors are shown in monomer 2. These cofactors are: the Mn-cluster (collection of red, purple and green spheres), tyrosine-Z (Y_Z_) (black sticks), P680 (green sticks), Pheophytin (yellow sticks), Q_A_ (red sticks), Q_B_ (blue sticks) and the non-heme iron (orange sphere). **(B)** Bicarbonate (BCT) binding to the non-heme iron (NHI) located between Q_A_ and Q_B_ on the acceptor side of PS II. Coordinates were taken from the *Arabidopsis thaliana* control structure (9I4T). Residues and cofactors are shown as blue sticks with red and blue sections indicating oxygen and nitrogen atoms, respectively. Dashed black lines indicated bonds linking the iron-quinone-bicarbonate complex **(C)** Release of bicarbonate during high light conditions. A dashed black arrow indicates the dissociation of the bicarbonate from the non-heme iron (NHI). Q_A_^•−^ is indicated with a blue circle representing an electron. Coordinates were taken from the current high light structure

Photosystem II (PS II) is a large protein complex found in the thylakoid membranes of photosynthetic organisms. PS II uses light energy to extract electrons from water, initiating photosynthesis, while also producing oxygen as a byproduct. PS II uses light energy to induce a charge separation between specially arranged chlorophyll molecules (named P680) and a pheophytin molecule (Pheo) found in the core of PS II. Pheo^•^^−^ then transfers the electron to a bound quinone molecule (Q_A_) on the ‘acceptor side’ of PS II and further to an exchangeable quinone (Q_B_). The positive charge on P680^•+^ is neutralized by an electron from a nearby redox active tyrosine (Y_Z_), which is in turn reduced by an electron from an oxo-bridged manganese, calcium cluster (Mn_4_CaO_5_, referred to hereafter as ‘Mn-cluster’) on the ‘donor side’ of PS II (Holzwarth et al. 2006; Renger and Renger 2008; Shen 2015; Kern et al. 2018; Bhowmick et al. 2023; Shevela et al. 2023; Umena et al. 2011) (Fig. 1A). After accepting two electrons and two protons, Q_B_ is released from the acceptor side of PS II as plastoquinol (Q_B_H_2_) and is replaced by an oxidized plastoquinone from the shared pool of plastoquinone in the thylakoid membrane (Kato et al. 2024; Saito et al. 2013; Van Eerden et al. 2017). On the donor side, the Mn-cluster sequentially donates 4 electrons before it oxidizes two ligated and deprotonated water molecules to form O_2_, while the electrons liberated during water oxidation restore the Mn-cluster back to its lowest (S_0_) oxidation state (Kok et al. 1970; see Shevela et al. 2023 for review).

During summer months, plants frequently operate in high light and high temperatures. These conditions can cause damage to PS II. High light accelerates the rate of PS II turnover leading to full reduction of the pools of electron carrier molecules, such as plastoquinone. This results in extended lifetimes of highly reactive redox intermediates such as Y_Z_^•^ or Q_A_^•−^, increasing the likelihood for side reactions that can damage PS II, either through direct damage, or through the production of radical oxygen species (ROS) (Krieger-Liszkay et al. 2008; Pospíšil 2009; Yamamoto et al. 2008). Excess light can also cause direct damage to the Mn-cluster, particularly blue light (Ohad et al. 2011; Ohnishi et al. 2005). Similarly, high temperatures can negatively affect photosynthesis, with PS II being particularly susceptible to heat stress (Busheva et al. 2012; Komayama et al. 2007; Yamamoto 2016; Yamamoto et al. 2008). Elevated temperatures can affect the fluidity of the thylakoid membrane, causing loss of the extrinsic subunits that help to enclose the Mn-cluster, leading to the loss of the Mn-cluster from PS II (Yamamoto 2016). Furthermore, lipid peroxidation is accelerated at high light and high temperatures(Chan et al. 2012; Pospíšil and Yamamoto 2017; Yamamoto 2016).

The most frequently damaged subunit of PS II is the D1 subunit (Greenberg et al. 1987), which provides ligands to the Mn-cluster, P680 and the redox active cofactors Pheo and Q_B_. Thus, photosynthetic organisms have developed a repair cycle in which PS II is disassembled, allowing the damaged D1 subunit to be degraded by FtsH and Deg proteases, before PS II is re-assembled around a newly synthesized D1 subunit (Komenda et al. 2010; Yoshioka and Yamamoto 2011). Previous studies have shown that in plants, one of the first steps of D1 degradation following photodamage is the cleavage of D1 into a 23 kDa fragment, and a 9 kDa fragment (Aro et al. 1993; Nakajima et al. 1996). It is hypothesized that this allows degradation of the damaged D1 protein as the FtsH proteases in plants are endopeptidases and cannot begin digestion from the N- or C-terminal (Yamamoto et al. 2008; Yoshioka and Yamamoto 2011). The site of D1 cleavage for this fractionation has been narrowed down to the loop connecting the D and E helices of the D1 protein, known as the DE loop (Aro et al. 1993; Greenberg et al. 1987). The cleavage of D1 can occur in isolated PS II complexes, indicating that this proteolysis event is auto-catalytic as it does not require any accessory proteases (Shipton and Barber 1992; Virgin et al. 1991).

Photosynthetic organisms have developed strategies to reduce PS II damage in high light conditions. These strategies include but are not limited to: (i) the rearrangement of the thylakoid membranes to better disperse excess light energy (Grinzato et al. 2020; Kouřil et al. 2013), (ii) the disassembly of antenna complexes to reduce the amount of energy passed to PS II (Fristedt and Vener 2011), (iii) non-photochemical quenching (NPQ) (reviewed in Zuo 2025), and (iv) the bicarbonate-mediated photoprotective effect on the acceptor side of PS II (Brinkert et al. 2016). This regulatory bicarbonate is a bidentate ligand of the non-heme iron (NHI), which is located on the acceptor side of PSII between Q_A_ and Q_B_ (Umena et al. 2011; Wydrzynski and Govindjee 1975) (Fig. 1B). In high light conditions when the plastoquinone pool is fully reduced, long-lived Q_A_^•−^ affects the binding strength of the bicarbonate to the non-heme iron, resulting in the dissociation of the bicarbonate ion (Fig. 1C) (Brinkert et al. 2016; Shevela et al. 2020). The effect of the bicarbonate dissociation is to change the redox potential of Q_A_/Q_A_^•−^, so that charge recombination through the singlet oxygen producing pathway has a lower probability, providing a photoprotective effect (Brinkert et al. 2016).

Our aim is to gain structural insight into the photoprotection and photodamage processes in PSII. Here, we present a first, exploratory study, in which we expose isolated PS II complexes to strongly damaging light/temperature conditions and determine the average structure of damaged PS II complexes using cryo-electron microscopy (cryo-EM). As the PS II complexes were illuminated after isolation, proteases were largely absent so that damaged PS II complexes remained undigested, facilitating the identification of areas of frequent damage.

## Methods

### Photosystem II extraction, purification and activity assay

*Arabidopsis thaliana* (Columbia-0) plants were grown for 8 weeks at constant temperature (20 °C) and humidity (70%), using day/night periods of 8 h light/16 h at a light intensity of approximately 125 µmol (photons) m^− 2^ s^− 1^ using full-spectrum, white light LED lamps (Ra 95; LEDlife 50 W Philipps; see Forsman et al. 2026 for details). The leaves of the plants were harvested after 16 h of darkness, and BBY membranes (PS II-enriched thylakoid membrane fragments, Berthold et al. 1981) and PSII complexes (isolated PS II in complex with the strongly bound light-harvesting antenna; C2S2) were isolated as described previously (Forsman et al. 2026).

### Exposure to high light intensities and electron microscopy

PSII complexes were concentrated and washed several times with buffer (50 mM MES pH 6.3, 20 mM CaCl_2_, 5 mM MgCl_2_, 0.6 M Betaine, 0.03% (w/v) β-DM) using 100 kDa molecular weight cut-off spin filters (Amicon). The sample (1.6 mg Chl mL^− 1^) was then put into a glass vial (approx. diameter 10 mm; sample height 10 mm) and exposed to 2000 µE of light for 30 min, provided by a projector, with a yellow filter to remove the blue part of the spectrum (Fig. S1). The tube was illuminated from the bottom and shaken regularly to provide mixing. Temperature changes during this-high light treatment were measured using TMD-50 K-type thermometer (Amprobe, U.S.A) (Fig. S2).

Approximately 30 s after turning off the high light, 4 µL of sample was loaded onto glow-discharged (30s at 50 mA) Quantifoil grids R 1.2/1.3 Cu 300 (Quantifoil Micro Tools) in dim light. The grids were then plunge frozen in liquid ethane using an FEI Vitrobot MkIV (Thermo Fisher Scientific) at 100% humidity, 4 °C, a blot force of -5, a wait time of 1 s and a blotting time of 5 s. Automated data collection was performed using EPU software on a Titan Krios G2 transmission electron microscope operating at 300 keV (Thermo Fisher Scientific) at the Umeå Core Facility for Electron Microscopy, a node of the Swedish National Cryo-EM facility. The Titan Krios was equipped with Falcon 4 direct electron detector and a Selectris energy filter. In total 15,511 movies were collected, which each contained 40 frames. The movies were collected at a pixel size of 0.7155 Å, a total dose of 40 electrons/Å^2^ and defocus values that ranged from − 0.8 to -2.0.

### Data processing

The dataset was processed using cryoSPARC software (Punjani et al. 2017). Motion and contrast transfer function corrections were performed before template free picking was used to identify particles. 2,002,863 particles were initially identified, before several rounds of 2D classification reduced the number of “good” particles to 111,992. 2D classification did not show any obvious heterogeneity in the number of antenna complexes bound to PS II, with all PS II complexes lacking the bound antenna (Fig. S3). Ab initio 3D reconstruction was used to generate 2 different 3D volumes, which were then refined using heterogeneous refinement. The particles used to generate the best-resolved class went through additional filtering using 2D classification and multi-class ab initio 3D reconstruction until a final volume was generated using 57,560 particles and refined using homogeneous refinement and non-uniform refinement with imposed C2 symmetry. The final volume had an estimated global resolution of 2.74 Å, using the gold standard FSC (Fig. S4). Finally, the map was sharpened, using Phenix to perform local sharpening with the Autosharpen function (Liebschner et al. 2019). CCP4 software was used for the low-pass filtering of the control map from 2.44 Å to 2.74 Å global resolution (Winn et al. 2011).

### Model building

The model of PS II from the control *Arabidopsis thaliana* that was frozen under dim light conditions (9I4T; Forsman et al. 2026) was used as a starting point and manually fitted into the map using UCSF ChimeraX software (version 1.7) (Meng et al. 2023). UCSF ChimeraX software was used to delete subunits from 9I4T which had no map density in the high light map. COOT (version 0.9.8.92) (Emsley et al. 2010) was used to manually refine the structure into the sharpened map. Several rounds of automatic fitting and manual fitting/checking were performed to optimize the fitting of the model into the map densities. Final refinement was performed using the Real-space refinement function in Phenix (Afonine et al. 2018) with manual checking in COOT to confirm or correct the automatic refinement. All structures in the figures were prepared with ChimeraX. Details of the data collection and model building are shown in Supplementary Tables 1–3.

### Water channel calculations

The channels to the exterior of the protein were calculated using CAVER software (Chovancova et al. 2012) (version 3.0.3), which is a plugin for PyMOL (Schrodinger and Delano 2020). For all channels, standard settings were used: probe radius 0.9, shell depth 4, shell radius 3, clustering threshold 3.5.

### OccuPy analysis

The occupancy of the different areas of the potential map was estimated using the software OccuPy (Forsberg et al. 2023). Standard settings were used and the post-processing sigmoidal option was selected to remove the signal from the solvent.

### Oxygen evolution

Oxygen evolution rates were measured using a Clark-type oxygen electrode (Hansatech, UK). Solubilized PS II complexes were diluted to 10 µg Chl mL^− 1^ with oxygen electrode buffer (10 mM MES pH 6.5, 200 mM Sucrose, 10 mM NaCl, 5 mM MgCl_2_). 20 µM phenyl-para-benzo-quinone (PPBQ) and 15 mM FeCN_6_ were used as artificial electron acceptors.

### Blue-Native PAGE

10 µL of isolated PS II complexes (1 mg Chl mL^− 1^) were taken at each timepoint in the high light treatment. 2 uL of loading buffer (50 mM Bis-Tris pH 7.0, 1 M 6-aminocaproic acid, 20% (v/v) glycerol, and 0.1% Coomassie Brilliant Blue G-250) was added to each aliquot and the PS II complexes were loaded onto the BN-PAGE gel (12 µL in each lane). The gel was run at 80 V for 2 h in Anode buffer (5 mM Bis-Tris, 5 mM Tricine pH 6.8) and dark blue cathode buffer (5 mM Bis-Tris, 5 mM Tricine pH 6.8, 0.02% Coomassie Brilliant Blue G-250), before changing the cathode buffer to light blue cathode buffer (5 mM Bis-Tris, 5 mM Tricine pH 6.8, 0.002% Coomassie Brilliant Blue G-250) and running at 150 V for an additional 6 h.

## Results

### High light treatment and cryo-EM data collection

The aim of this exploratory study was to observe damage to PS II following high light/elevated temperature conditions. Therefore, extreme conditions were chosen to maximize the opportunity to observe various types of damage. Solubilized PS II complexes were exposed to 2000 µE of light for 30 min, which resulted in a temperature increase to 29 °C (Fig. S2). 2000 µE was chosen as this is approximately the maximum solar radiation experienced in nature i.e. middle of the day in summer. As elevated temperatures often accompany high light conditions, the temperature was allowed to increase during the high light treatment. Experiments on solubilised PS II complexes showed that 30 min of high light/elevated temperature treatment would give the best chance of observing uniform damage in the final structure, as this duration resulted in approximately 75% loss of oxygen activity (Fig. S5A), indicating damage, while still maintaining sufficient intact PS II complexes to allow for a high-resolution structure to be calculated (Fig. S5B). PS II complexes were extracted from the thylakoids before the treatment so that the damaged PS II complexes could be quickly frozen, allowing the damaged PS II complexes to be captured by cryo-electron microscopy (cryo-EM).

**Fig. 2:**
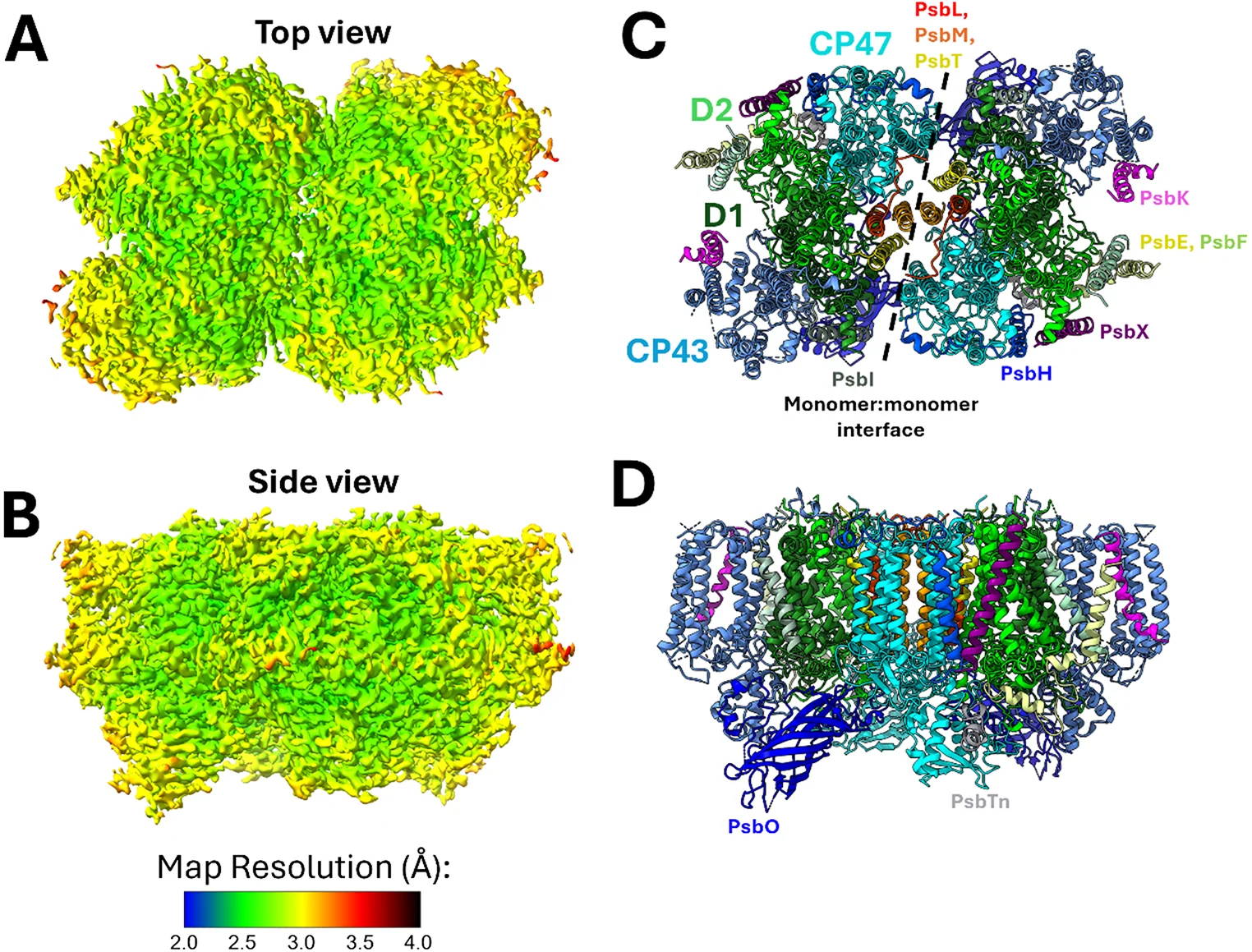
Structure of photosystem II core complexes from *Arabidopsis thaliana* after treatment with high light. Top view **(A)** and side view **(B)** of the Coulomb potential map, which is coloured to represent the local resolution. Top view **(C)** and side view **(D)** of the model which was built into the high-light PS II map. The different subunits are shown in different colours and labelled in the corresponding colour. Due to space limitations the labels for the PsbL, PsbM and PsbT subunits, which are all located in the centre of the dimer interface, are shown on top of the dimer. Each subunit is only labelled in one of the monomers

Cryo-EM (single particle analysis) of the high light treated PS II complexes captured 57,560 high quality particles which generated a Coulomb potential map with a global resolution of 2.74 Å (Fig. S4), ranging from approximately 2.5 Å resolution in the core to approximately 3.0 Å in the peripheral regions (Fig. 2A-B). Using this map, a model was built to represent the average PS II structure following high light/elevated temperature treatment, known hereafter as the “high light structure” (Fig. 2C-D). The PS II complexes used in this study were handled in the same way as in the control *Arabidopsis thaliana* PS II structure (9I4T) (Forsman et al. 2026), with the only difference being the high light/elevated temperature treatment.

### Loss of subunits

**Fig. 3:**
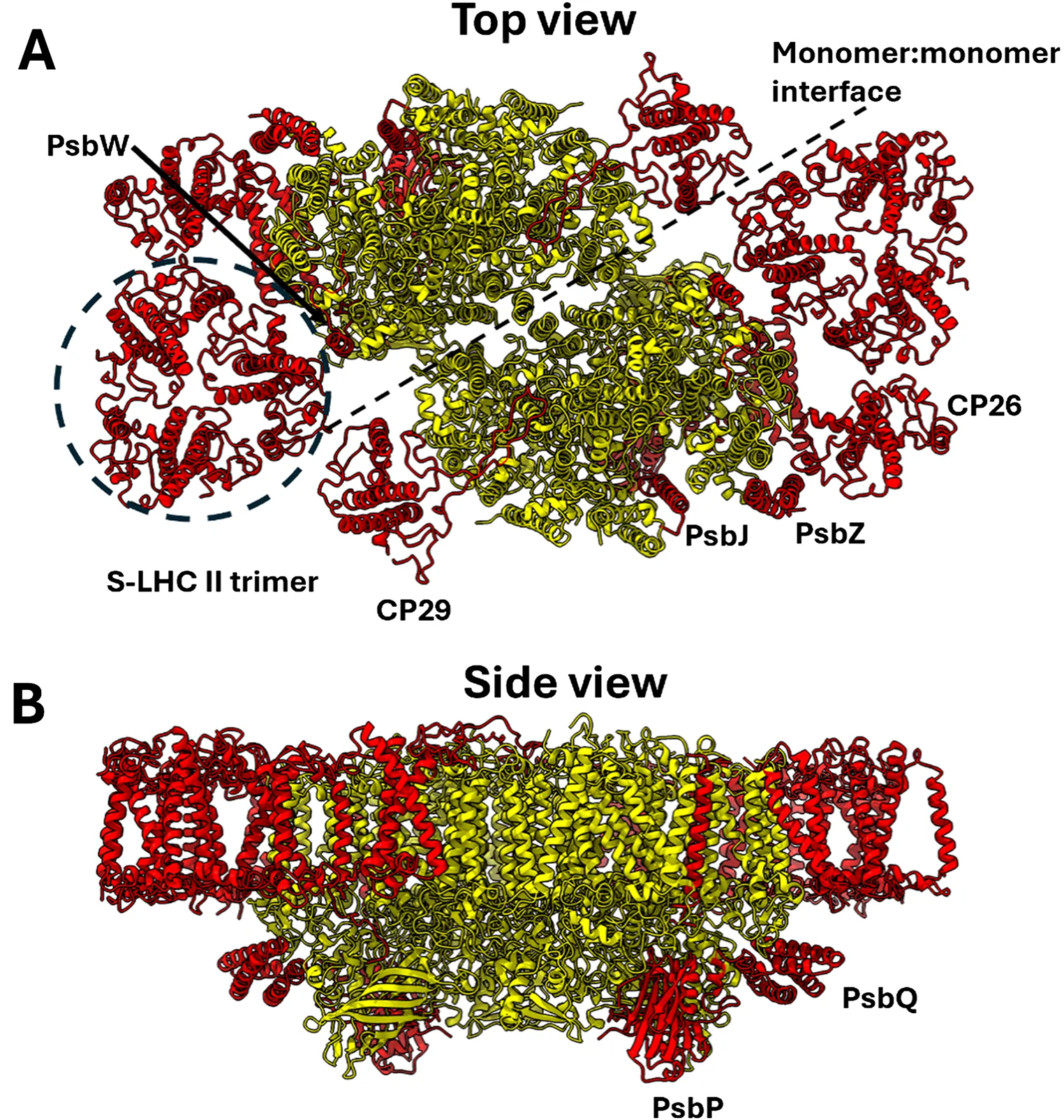
Missing subunits in the high-light PS II structure. The high-light structure is shown in yellow. Subunits which are present only in the control structure (9I4T) are shown in red and are labelled in only one of the monomers. The subunits missing in the high-light PS II complex are: CP26, CP29, PsbJ, PsbP, PsbQ, PsbW and PsbZ, along with the strongly bound-LHC II trimer (S-LHC II trimer). **(A)** top and **(B)** side view

The Coulomb potential map for the high light/elevated temperature treated PS II complexes showed no density for the strongly bound LHC II trimer antenna or the CP26 and CP29 linker subunits, indicating that these subunits had been lost during treatment in the majority of the PS II complexes. Additionally, the small transmembrane PsbJ, PsbW and PsbZ subunits as well as the extrinsic PsbP and PsbQ subunits showed no density in the map (Fig. 3). Despite the loss of the PsbP and PsbQ subunits, the Mn-cluster was still largely present. When the Coulomb potential map for the control was low-pass filtered to 2.74 Å to match the global resolution of the high light map, the Coulomb density around the Mn-cluster was comparable in both the dim and high light maps (Fig. S6), indicating similar occupancy for the Mn-cluster.

The relative abundance of the remaining subunits that make up the high light treated PS II complexes was evaluated using the software OccuPy (Forsberg et al. 2023). Relative to the CP47 subunit, CP43 was estimated to have approximately 90% occupancy, while PsbO showed approximately 75% occupancy (Fig. S7). The CP43 subunit has several loops facing the exterior which cannot be resolved. This is likely due to increased flexibility caused by loss of subunits which typically bind to the surface of CP43, for example CP26 or the light harvesting antenna complex.

### Sites of photodamage

The cryo-EM map represents a consensus map of the average structure of PS II following the high light/elevated temperature treatment. Consequently, it cannot be used to identify the precise sequence and/or details of how individual PS II complexes were damaged; however, frequently damaged areas can be identified by structural changes or reduced resolution relative to their surroundings, caused by damage-induced variability. Sites of interest are those surrounding: (i) the reaction center pigments and their environment, which includes P680 where ^1^O_2_ formation is proposed to occur through interaction with triplet chlorophyll formed by charge recombination reactions (Krieger-Liszkay et al. 2008), (ii) the environment around the Mn-cluster, which may be damaged by ROS generated by incomplete water oxidation (Wydrzynski et al. 1996) and (iii) the environment around the non-heme iron on the acceptor side, which is a proposed site of superoxide formation (Fantuzzi et al. 2022) and bicarbonate mediated photoprotection (Brinkert et al. 2016) as well as D1 auto-cleavage (E.-M. Aro et al. 1993; Nakajima et al. 1996). The Coulomb potential map was carefully inspected to determine if these areas exhibited reduced resolution, indirectly indicating damage, or specific resolved differences to the control structure (9I4T) indicating a structural response to the high light treatment. It should be noted that C2-symmetry was enforced in the generation of the cryo-EM map, therefore asymmetric damage would be averaged out across both monomers.

#### Environment of the reaction center pigments

In the high light structure, there were no obvious signs of damage around Pheo or the P680 chlorophylls. The pheophytin and chlorophyll molecules as well as the surrounding residues were very similar in the control and the high light structures, with only minor movements of residue or cofactor positions and no loss of resolution (Fig. S8).

**Fig. 4:**
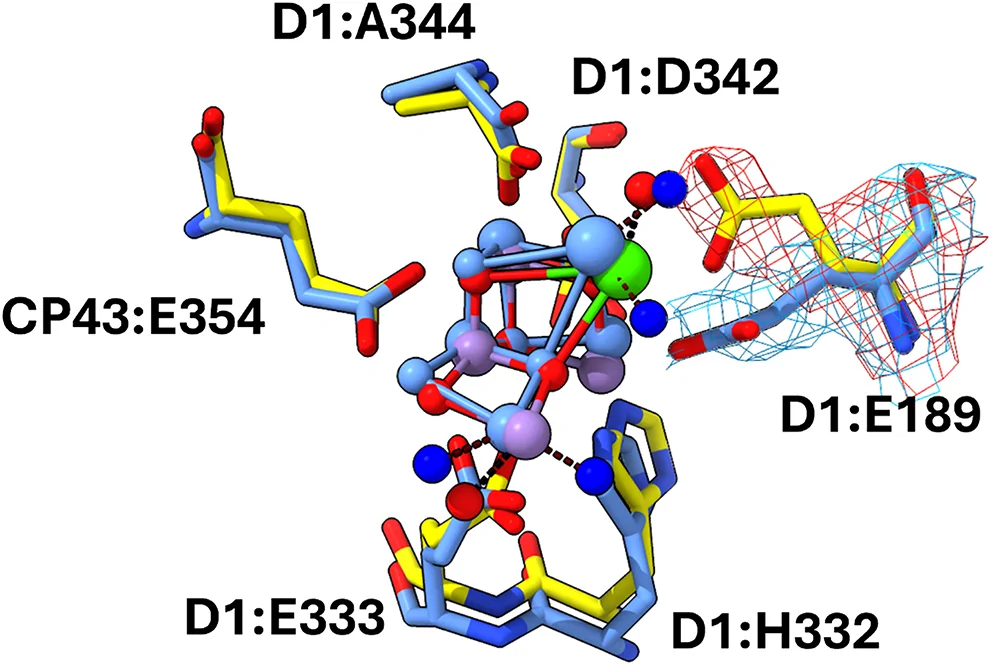
Comparison of the Mn-cluster in the control (dim light) and high-light PS II structures. The Mn-cluster and ligating residues of the control structure are shown in light blue and those of the high-light structure are shown in yellow, with nitrogen and oxygen atoms (excluding Mn-cluster oxygens) shown in red and dark blue, respectively. For the high-light structure the Mn-cluster is shown as a group of red (oxygen), purple (manganese) and green (calcium) spheres. The D1:E189 residue shows a considerable difference between the two structures, so the Coulomb potential maps around the residue are shown as a blue mesh (control map) or red mesh (high-light map) to demonstrate this residue is modelled correctly into the respective maps

#### Environment around the Mn cluster

The most obvious difference in the protein environment of the Mn-cluster between the control and high light PS II was the conformation of the D1:Glu189 residue. Under normal conditions, the D1:Glu189 residue ligates the Mn1 atom of the Mn-cluster. In the high light structure, the D1:Glu189 residue has moved upwards, such that it is no longer within ligation distance of the Mn1 atom (Fig. 4). All other ligands to the Mn-cluster were in comparable positions in both the control and high light structures, indicating that position of D1:Glu189 may be particularly sensitive to damage in the OEC.

Since cryo-EM data collection damages the Mn-cluster by reducing the metals, causing the bond lengths to extend (Forsman et al. 2026; Hussein et al. 2024; Kato et al. 2021), the differences between the positions of the metal ions of the Mn-cluster in the high light and control structures were not analysed as they were unlikely to be physiologically relevant. The redox active tyrosine’s Y_Z_ (D1:Tyr161) and Y_D_ (D2:Tyr160) looked similar in the control and high light structures, as well as the Cl1 and Cl2 chloride ions (Fig. S9).

#### Environment around the non-heme iron

**Fig. 5:**
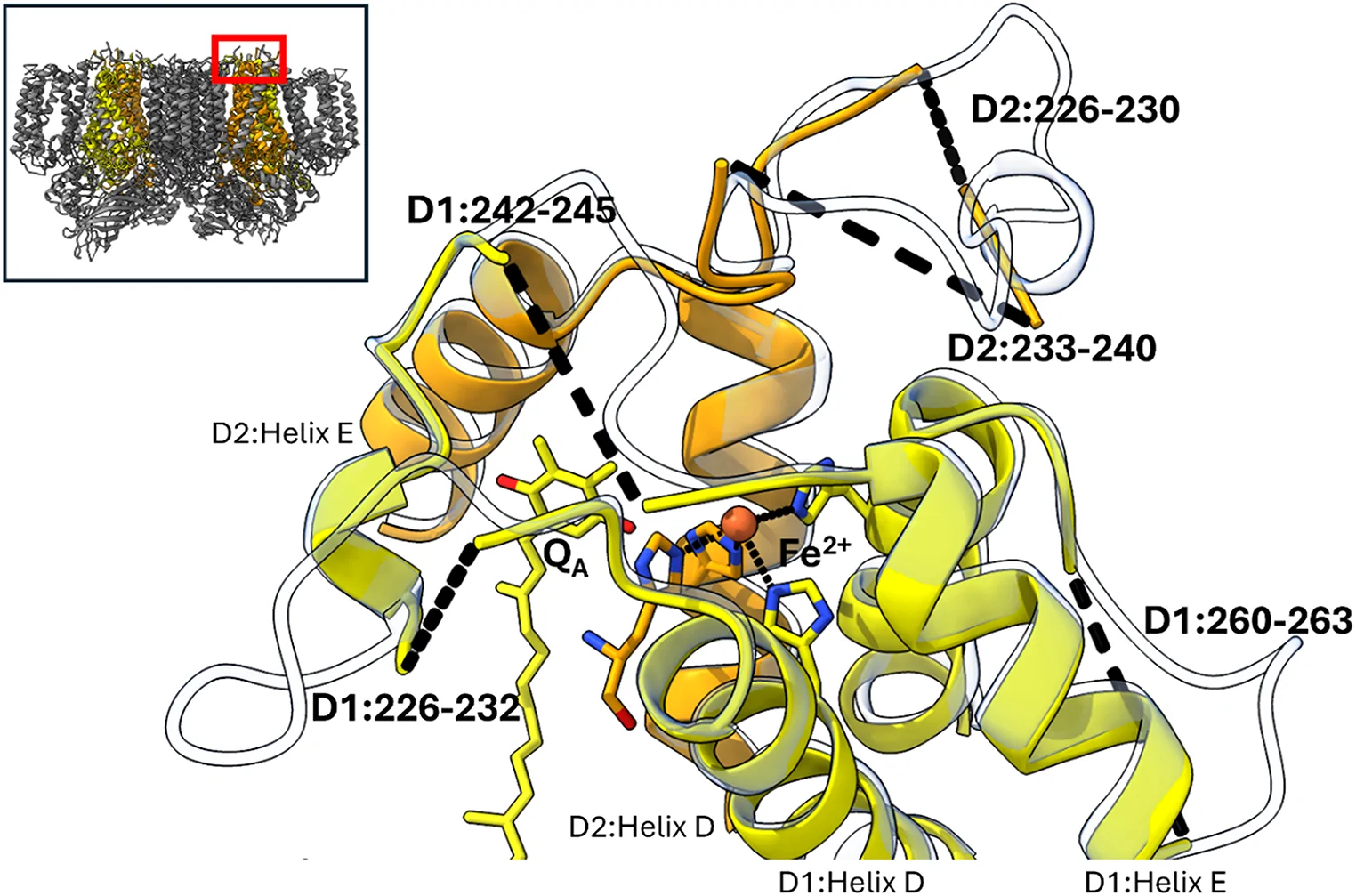
Modelling of the D1 and D2 DE-loops in the high-light structure. The insert in the top left shows the location of the DE loops for D1 and D2, with a red box highlighting the area shown in the rest of the figure. D1 is shown in yellow, D2 is shown in light orange. The D1 and D2 subunits of the control structure (9I4T) are shown as faint white ribbons. Regions which could not be modelled due to lack of Coulomb potential density are shown as thick dashed lines. The residues which could not be modelled are labelled beside the dashed lines. Q_A_ and the histidine ligands to the non-heme iron are also shown as yellow or orange sticks. The non-heme iron is shown as a dark orange sphere. The D and E helices of D1 and D2 are labelled

On the acceptor side, there was a loss of Coulomb potential density at specific loop regions of the D1 and D2 subunits (Fig. S10). The D1:226–232, D1:242–245, D2:226–230 and D2:233–240 loops showed little/no density in the Coulomb map after the high light treatment (Fig. 5), while the rest of the D1 and D2 subunits were well resolved (for the D1:260–264 loop, see next section). The loss of electron density in these regions indicates damage or temperature induced structural variability in these loop regions. Channels connecting the non-heme iron to the protein exterior were calculated in the high light treated PS II structure to determine if the loss of density in the loop regions corresponded to the exits of the channels (Fig S10). This was to investigate if ROS produced at the non-heme iron could diffuse through the channels and damage loop regions near the channel exits. While two regions of lost density were located near the exit of Channel A (acceptor side channels discussed in Sirohiwal and Pantazis 2022), the other two regions of lost density were not close to the exit of either Channel A or Channel B (Fig. S11).

**Fig. 6:**
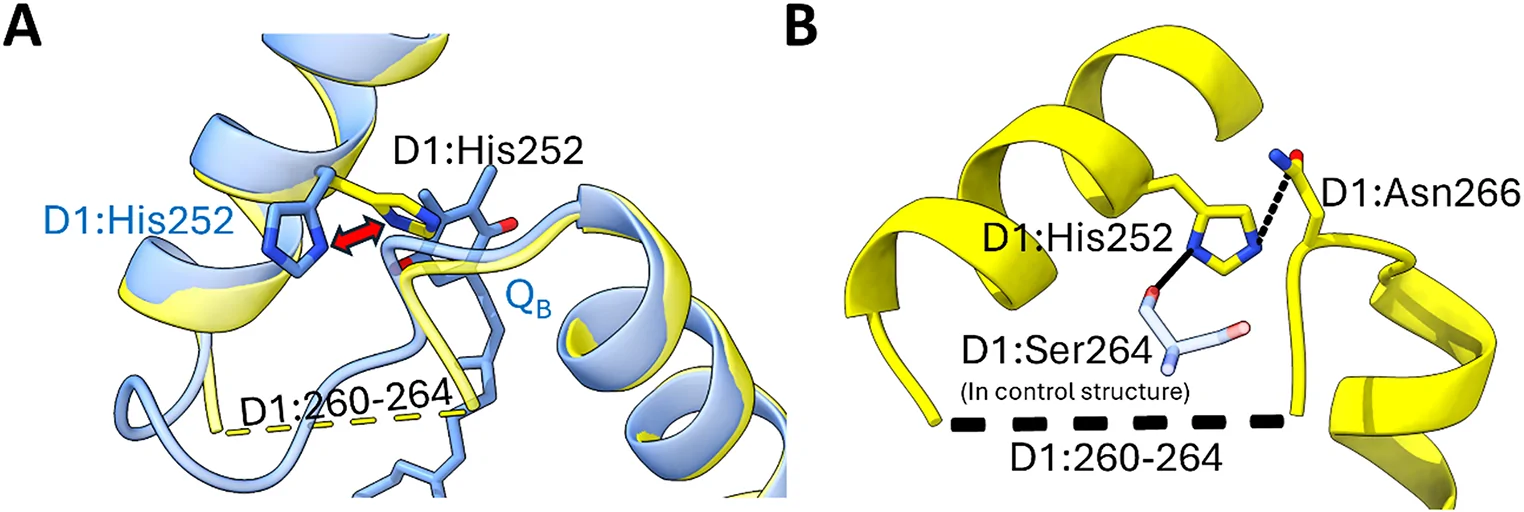
Movement of the D1:His252 residue in the high-light PS II structure. **(A)** The Q_B_-binding pocket in the control structure (blue, 9I4T) and the high-light structure (yellow). The D1:His252 residue is shown as sticks in both structures with a red arrow indicating the movement of this residue between structures. In all cases, red and blue sections indicate oxygen or nitrogen atoms. Q_B_ is labelled in the control structure and the unmodelled D1:260–263 loop is labelled in the high-light structure. **(B)** Putative serine-protease triad in the high-light structure. The D1:His252 and D1:Asn266 residues are shown as yellow sticks. The D1:Ser264 residue is not resolved in the high-light structure, therefore, the D1:Ser264 residue from the control structure is shown in transparent blue (9I4T). Black dashes lines indicate the putative connections between the D1:Asn264-D1:His252 (4.2 Å) and D1:His252-D1:Ser264 (3.8 Å). The unmodelled D1:260–263 loop is shown as a thick dashed black line

A further area of reduced electron density is found in the D1:260–264 loop, located near the Q_B_-binding pocket. Interestingly, in the high light structure, the nearby D1:His252 residue is found rotated relative to the control so that it faces inwards into the empty Q_B_-binding pocket (Fig. 6A, Fig. S12). In the literature, there is evidence indicating that following damage, autocatalytic activity of a serine protease cleaves the D1 protein, somewhere in the DE loop (Aro et al. 1993; Greenberg et al. 1987; Nakajima et al. 1996). The movement of the D1:His252 residue brings it to within 4.2 Å of D1:Asn266 and 3.8 Å from where D1:Ser264 is located in the control (Fig. 6B). We speculate that this may form an atypical serine-protease triad or diad (Ekici et al. 2008), leading to auto-cleavage of the D1 protein (Fig. S13).

**Fig. 7:**
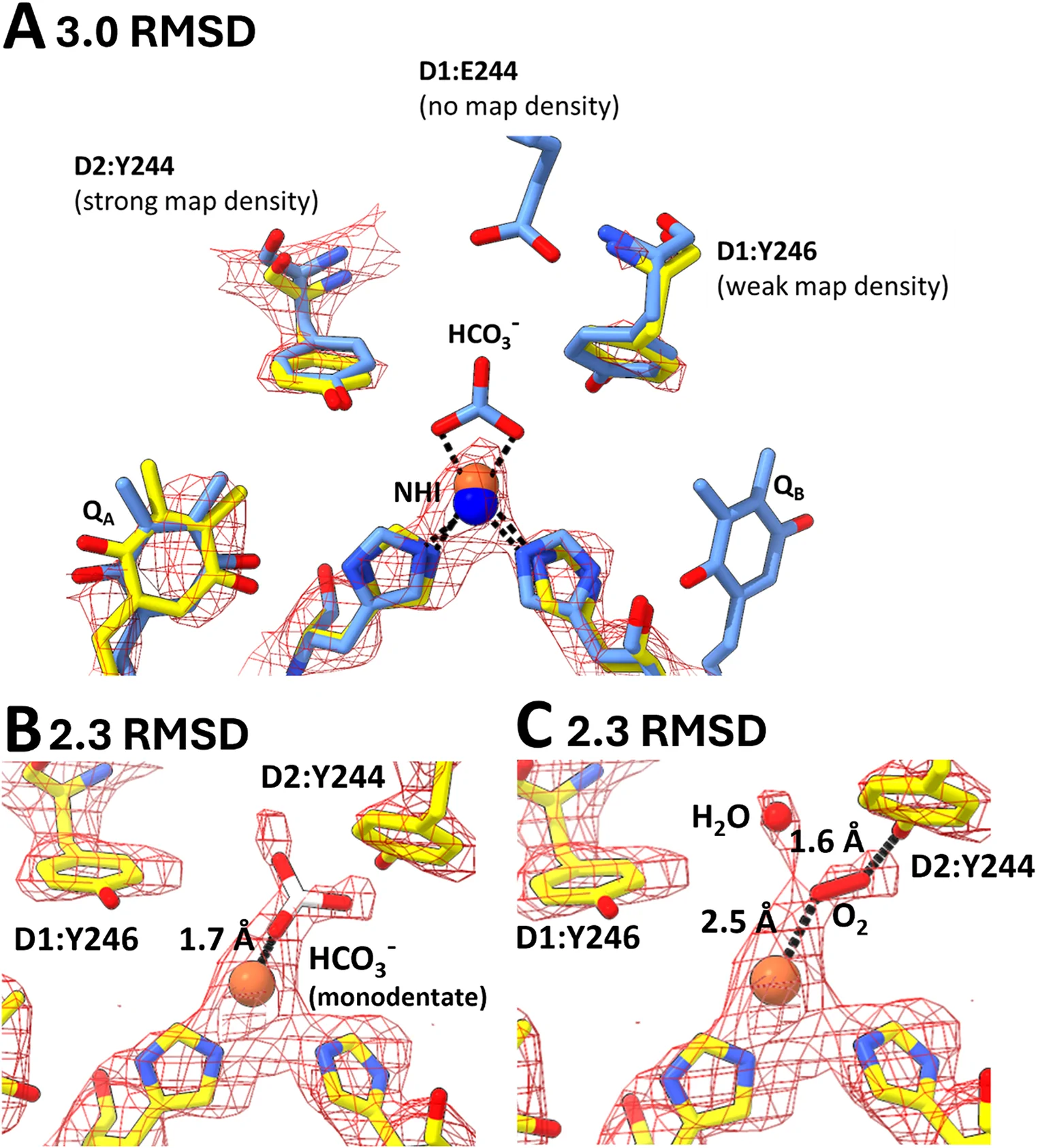
**(A)** Comparison of the non-heme iron (NHI) environments on the acceptor sides of PS II in the control and high-light structures. The control structure (9I4T) is shown in light blue sticks, the high-light structure is shown in yellow sticks. Red and dark blue sections indicate, oxygen and nitrogen atoms, respectively. The NHI is shown as a dark blue sphere (control) or an orange sphere (high-light structure). Important residues which appear to ligate to the bicarbonate ligand are shown and labelled. The Coulomb potential map of the high-light treated PS II at a contour level of 3.0 RMSD is shown as a red mesh to indicate correct modelling of the residues/cofactors. **(B)** Fitting of a monodentate bound bicarbonate ligand into the weak map density, which appears above the NHI at 2.3 RMSD contour level. The distance between the bicarbonate and the NHI in this model is 1.7 Å. The distance between the bicarbonate and the nearby D2:Tyr244 residue is 1.3 Å. **(C)** Fitting of an oxygen molecule ligand and a water into the weak map density at 2.3 RMSD contour level. The distance between the oxygen and the NHI is 2.5 Å, the distance between the oxygen and the D1:Tyr246 residue is 1.6 Å

The bicarbonate ligand to the non-heme iron has been proposed to act like an electrical fuse, un-binding from the iron in high light conditions to minimize damage by dampening activity, then rebinding when conditions return to normal, allowing full PS II activity to resume. Specifically, it was demonstrated previously that the absence of the bicarbonate ligand alters the midpoint potential of Q_A_/Q_A_^•−^, facilitating charge recombination reactions avoiding chlorophyl radical triplet state formation, thereby minimizing ROS production (Brinkert et al. 2016). At a contour level of 3.0 RMSD the Coulomb potential map for the high light treated PS II complexes showed no density above the non-heme iron, while there is a clear density at a comparable contour level for the bicarbonate in the Coulomb potential map of our control PS II structure (9I4T), indicating that the bicarbonate ligand is absent in the majority of the high light PSII complexes (Fig. 7A). To ensure that this loss of signal was not due to the lower resolution of the high light Coulomb potential map, relative to the control, the control map was reprocessed from 2.44 Å to 2.74 Å to allow direct comparison. This reprocessed control map still showed a clear density above the non-heme iron fitting the bicarbonate ligand (Fig. S14). Our result provides complementary structural information to the significant biophysical evidence that high light can cause the bicarbonate to dissociate from the non-heme iron (Brinkert et al. 2016; Shevela et al. 2012, 2020).

When the contour level is reduced to 2.3 RMSD, a weak density appears in the high light structure above the non-heme iron, suggesting that in a fraction of the high light treated PSII complexes there is an unidentified ligand bound to the non-heme iron. A bicarbonate bound in a bidentate orientation provides a poor fit for this density; however, a bicarbonate bound in a monodentate orientation fits this density reasonably well (Fig. 7B). It has been shown that, in the absence of bicarbonate, oxygen can bind to the non-heme iron and be reduced to superoxide (Fantuzzi et al. 2022). Therefore, we also tried modelling the unknown density above the non-heme iron with an oxygen and a water (Fig. 7C).

## Discussion

The most obvious effect of the high light/elevated temperature treatment on the structure of PS II was the loss of the antenna and linker subunits (Fig. 3). Previous studies have shown that elevated temperatures increase the fluidity of the thylakoid membranes, which can lead to the loss of subunits (reviewed in Yamamoto 2016). Typically, temperatures of 40 °C are required to observe loss of subunits from thylakoid-embedded PS II complexes (Busheva et al. 2012; Komayama et al. 2007; Yamamoto 2016; Yamamoto et al. 2008), however, as the solubilized PS II complexes used in this study have relatively thin detergent belts, it is probable that subunit loss can occur at lower temperatures than in thylakoid membranes. The removal of the antenna subunits has been suggested to be beneficial to PS II complexes in high light conditions as it would reduce the amount of excess light energy being passed to P680 (Kouřil et al. 2013). In vivo, active separation of the antenna complexes from the PS II core requires phosphorylation by the accessory protein STN7 (Fristedt and Vener 2011), which cannot take place in isolated PS II particles. Consequently, in this situation, it is more likely that the antenna subunits were lost passively at the elevated temperatures (Fig S2.) due to increased membrane fluidity.

The PsbJ, PsbW and PsbZ small transmembrane subunits and the PsbP and PsbQ extrinsic subunits are also absent in the high light structure, indicating that these subunits have weaker binding than the other core subunits (Fig. 3). In addition, the CP43 and PsbO proteins displayed reduced occupancy compared to CP47 (Fig. S7). During the repair cycle, the CP43 and PsbO subunits are released from the damaged PS II complex, to allow the damaged D1 subunit to be replaced by a newly synthesized D1 (Aro et al. 2005; Li et al. 2024a, b; Su et al. 2024). Therefore, they may be expected to be less tightly bound to the PS II complex than other PS II core subunits, in line with these observations.

Surprisingly, no clear sign for damage was found near P680, although it is expected to be a site for ROS formation via triplet states created during charge recombination. We suggest that our present resolution may not be high enough to resolve amino acid modifications due to ROS unless the majority of the PSII complexes are affected in the same way. ROS may instead attack the protein in a non-specific way due to the high reactivity of singlet oxygen, blurring out the effect, while at the same time the tight packing in the core prevents larger structural changes.

By contrast, the environment around the Mn-cluster did show a clear difference in the high light structure with the D1:Glu189 residue being in an unusual position. Notably, the high light map showed one clear density for the D1:Glu189 residue, rather than a loss of density which would have suggested that following the high light treatment the D1:Glu189 residue has a range of different conformations. The resolution of our present structure is not high enough to identify the precise cause for D1:Glu189 taking this new position. We note that D1:Glu189 residue is known to be more flexible than the other ligands to the Mn-cluster, as it has been shown to move during the S_2_-S_3_ transition, presumably facilitating the binding of a substrate water (Ibrahim et al. 2020; Isobe et al. 2024; Li et al. 2024a, b; Suga et al. 2019). At present, we cannot discern if the 25% O_2_ activity we observe (Fig. S5A) comes from a quarter of centres with D1:Glu189 in the regular position, or from a reduced activity of centres with D1:Glu189 in the ‘high light’ position. It is worth noting that this unusual position of D1:Glu189 is not observed in PS II structures which lack the Mn-cluster (Fig. S15) (7OUI, Graça et al. 2021; 5MX2, Zhang et al. 2017), indicating that this unusual position is due to photodamage, and not due to loss of the Mn-cluster in some complexes.

The region of the high light structure which showed the greatest difference to the control was the acceptor side of PS II, specifically the hydrophilic loops which connect the D and E loops in the D1 and D2 subunits (Fig. 5). Sections of these loops had insufficient density to be modelled (Fig. S10), despite good density for the rest of the D1 and D2 subunits. This may indicate damage-induced mobility in these loops, blurring the cryo-EM consensus map. Two regions of damage were located near the exit of Channel A (Fig. S11), indicating ROS formed on the acceptor side may pass through Channel A causing damage. However, this hypothesis is weakened by the observation that the other two un-resolved loops are not located near the exit of either of the acceptor side channels, suggesting that these loops may just be regions that are highly susceptible to damage induced structural flexibility.

In the high light structure, we observe loss of electron density for the D1:260–264 loop. Additionally, movement of D1:His252 forms a motif akin to a serine-protease triad. We, therefore, propose that damage on the acceptor side, accompanied by long-term absence of Q_B_, results in a conformational change at the Q_B_-binding site, whereby the D1:His252 residue is moved to face into the Q_B_-binding pocket (Fig. 6A). This completes an atypical serine-protease triad made up of D1:Ser264, D1:His252 and D1:Asn266 (Fig. 6B) that may facilitate auto-proteolysis of the D1 subunit, generating the 23 kDa and 9 kDa fragments which have been observed to appear following high light treatment (Aro et al. 1993). This hypothesis can explain: (i) why this D1 fragmentation can occur in isolated PS II complexes i.e. separated from any accessory proteases (Nakajima et al. 1996; Shipton and Barber 1992; Virgin et al. 1991), (ii) why serine-protease inhibitors can prevent this fragmentation (Shipton and Barber 1992; Virgin et al. 1991) and (iii) why this fragmentation has a similar pH dependence to the pKa of histidine (Nakajima et al. 1996). Furthermore, it has been shown that some herbicides which bind to the Q_B_-binding site prevent this fragmentation (Zer and Ohad 1995) and others cause the D1 fragmentation to occur in the dark (Nakajima et al. 1996), suggesting that some herbicides may prevent this D1:His252 movement, and others facilitate it. A previous cross-linking study identified the site of D1 cleavage being in the D1:226–244 range (Mizusawa et al. 2003), however, it is unlikely that the proposed serine-protease at the Q_B_-binding site would be able to cleave D1 in the D1:226–244 range which is 14–23 Å away (Fig. S16). We, therefore, suggest that the D1 subunit is cleaved in the D1:260–264 region, which is why this region is not resolved in the high light structure. This is also supported by mutagenesis studies which demonstrate that mutation of D1:His252 prevents the formation of damage induced crosslinking between D1 and cytochrome b-559, which may occur following D1 cleavage (Lupínková et al. 2002).

It is currently unclear from the high light structure if the proposed serine-protease triad would be capable of auto-catalytic cleavage of D1, as it has unusually long distances, an atypical asparagine component, and no clear target for nucleophilic attack (Du et al. 2025). However, our high light structure presumably represents the state of PS II following D1 cleavage. Therefore, we hypothesize that damage to the acceptor side of PS II, coupled with long-term vacancy of the Q_B_-pocket, generates a structure where the serine-protease triad is formed, which then, post-D1 cleavage relaxes into the observed high light structure.

In the high light structure, no density for bicarbonate was present at a contour level of 3.0 RMSD and only a weak density appeared at 2.3 RMSD. This supports the hypothesis that in high light conditions the bicarbonate ligand to the non-heme iron is driven off, providing a photoprotective effect (Brinkert et al. 2016; Shevela et al. 2012, 2020). The weak density observed at high contour levels is a poor fit for a bidentate bicarbonate ligand, however, a monodentate bicarbonate fits this density well, albeit with an unusually short (1.3 Å) bond to D2:Tyr244. A monodentate bound bicarbonate is in line with proposed desorption pathways (Sugo and Ishikita 2022). Considering that there was approximately 30 s of dim light conditions between the termination of the high light treatment and the freezing of the samples, re-binding of bicarbonate in a fraction of PS II complexes may occur. The unusual binding of the bicarbonate (monodentate, as opposed to bidentate) may also be caused by changes in the protein environment around the bicarbonate following the high light treatment. For example, the D1:Glu244 residue, which appears to stabilise the bicarbonate in the normal-light structure (9I4T); shows no density in the high light map (Fig. 7A), suggesting that following the high light treatment, this residue has high mobility. This is further supported by mutational studies, which have established that the binding of bicarbonate is prevented when the D1:Glu244 residue is mutated (Forsman et al. 2019). We also considered that the weak density above the non-heme iron may be due to oxygen binding to the non-heme iron. This has been shown to occur when the bicarbonate binding site is empty (Fantuzzi et al. 2022). However, this assignment also results in an unusually short distance between the oxygen and the nearby tyrosine D1:Tyr246 (Fig. 7C).

In summary, this in vitro study shows several interesting observations regarding photoprotection and photodamage of PSII. It thereby lays the basis for future, more detailed studies that may include various light and temperature conditions as well as exposure times. While this in vitro approach cannot resolve all mechanisms and interactions at play in vivo, it provides a new path towards observing structural consequences of photoprotection and photoinhibition, presently not accessible by in vivo approaches.

## Supplementary Information

Below is the link to the electronic supplementary material.

Supplementary Material 1

## Data Availability

Model and map data is deposited on pbd (https://www.rcsb.org/) under the accession code 31FD.
