# Supplementary material for: Effects of high light and elevated temperature on the structure of photosystem II isolated from Arabidopsis thaliana
Source: Photosynth Res. 2026 Oct 8;164(5):50. doi: 10.1007/s11120-026-01238-w (PMC13649839; doi:10.1007/s11120-026-01238-w)
Supplement: Supplementary file 1 [file 11120_2026_1238_MOESM1_ESM.pdf]

Supplementary Materials for

Effects of high-light and elevated temperature on the structure of Photosystem II isolated from *Arabidopsis thaliana*

Jack Forsman[1,2]*, André T. Graça[1,3], Michael Hall[1], Wolfgang P. Schröder[1,4]*, Johannes Messinger[2,4]*

[1]Department of Chemistry, Umeå University, SE 90187 Umeå, Sweden.

[2]Molecular Biomimetics, Department of Chemistry-Ångström, Uppsala University, SE 75120 Uppsala, Sweden.

[3]European Molecular Biology Laboratory, EMBL Grenoble, 38042 Grenoble, France

[4]Umeå Plant Science Center, Department of Plant Physiology, Umeå University, SE 90187 Umeå, Sweden.

*Corresponding Authors:

Jack Forsman: jack.forsman@umu.se

Wolfgang P. Schröder: wolfgang.schroder@umu.se

Johannes Messinger: johannes.messinger@umu.se

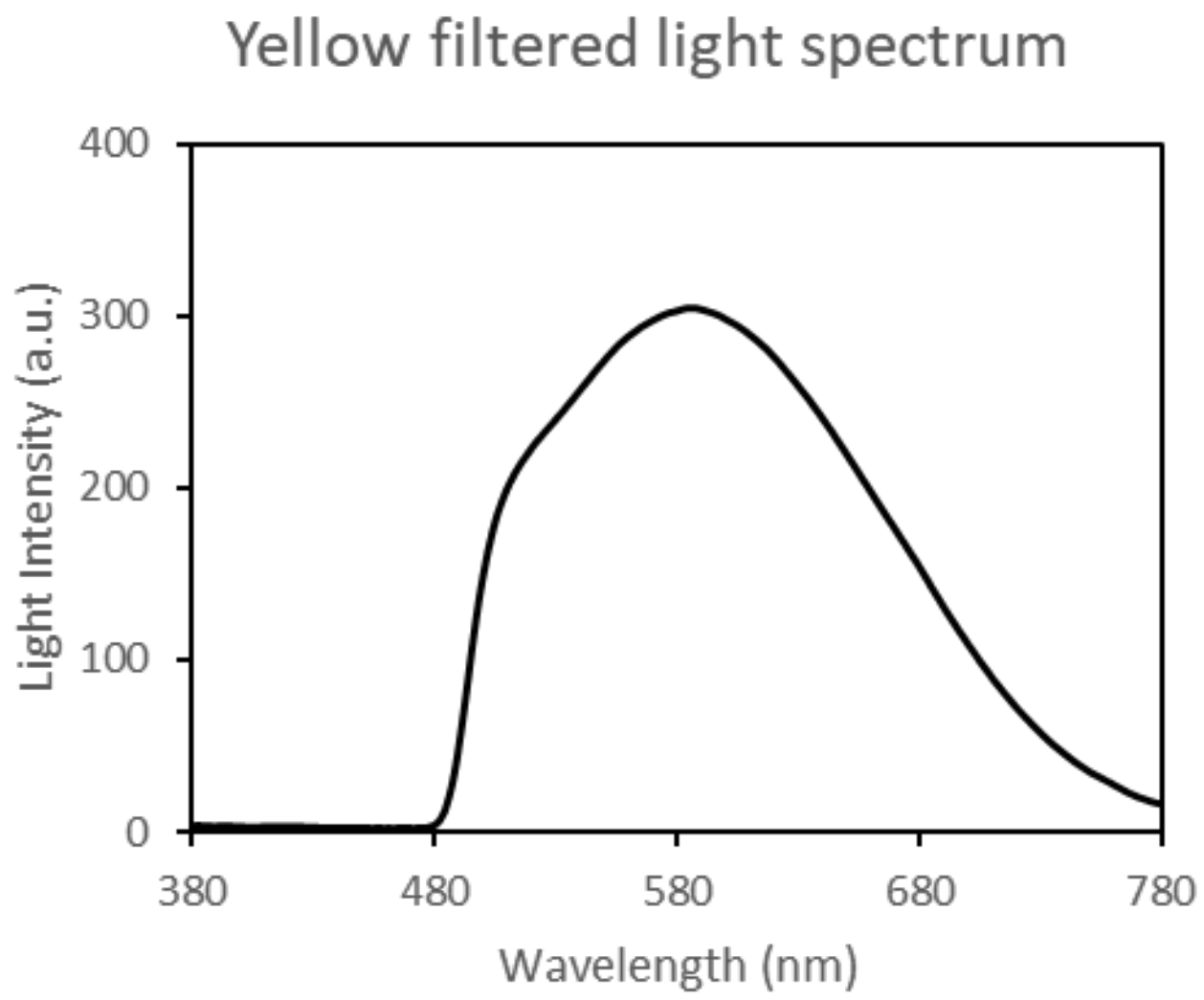

Supplementary Figure 1: Spectrum of the light used for the high-light treatment of the isolated Photosystem II complexes. This represents the average from 3 measurements. The light intensity is shown with arbitrary units (a.u.). The spectrum was measured with an LI-180 spectrophotometer (LI-COR, U.S.A).

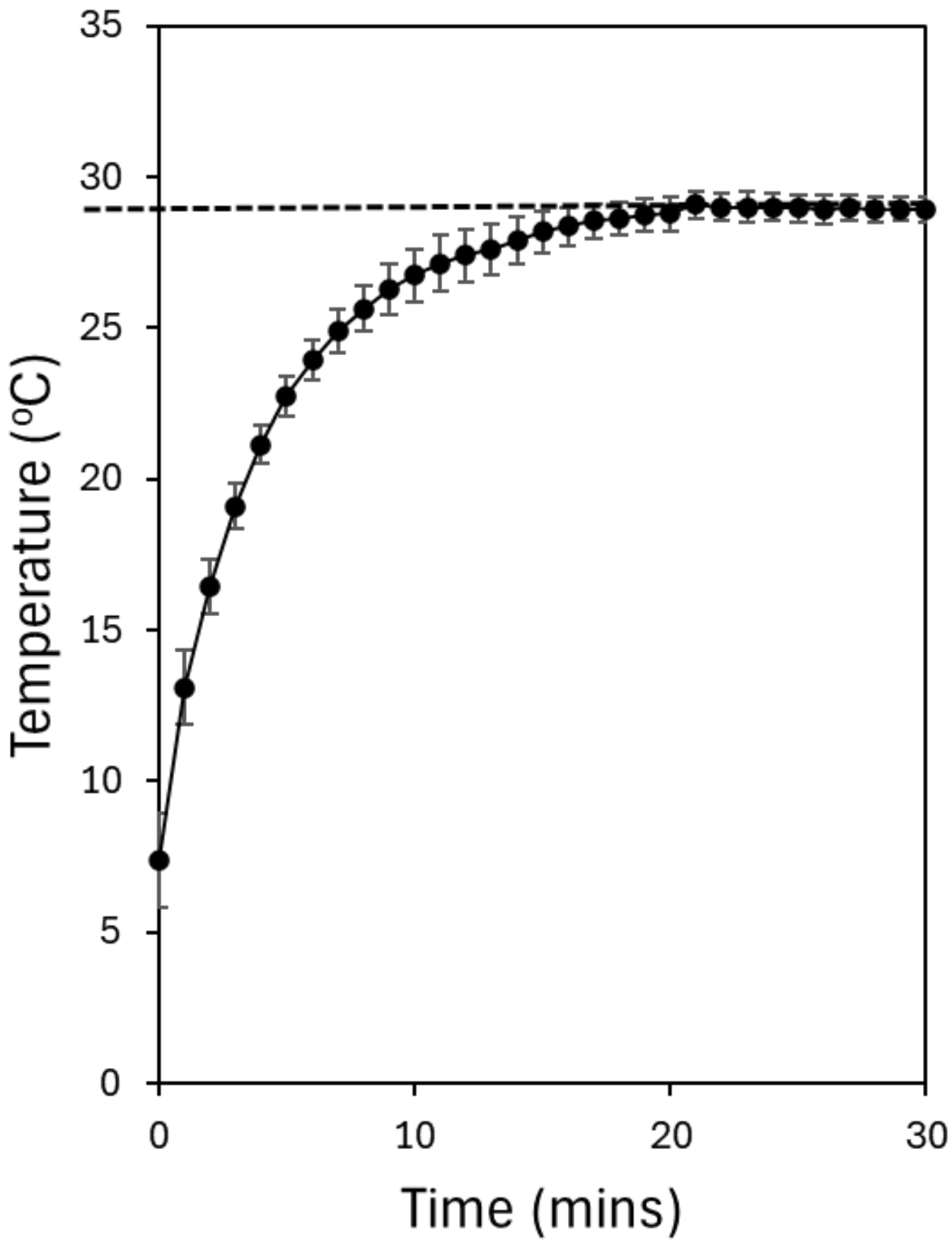

Supplementary Figure 2: Changes to the temperature of the solubilized PS II complexes during the high light treatment. Initially the complexes were kept on ice and warmed up during the high-light treatment. The graph shown represents the average of three repeats, with the error bars representing the standard error. The graph plateaus at 29°C which is indicated with a dotted line

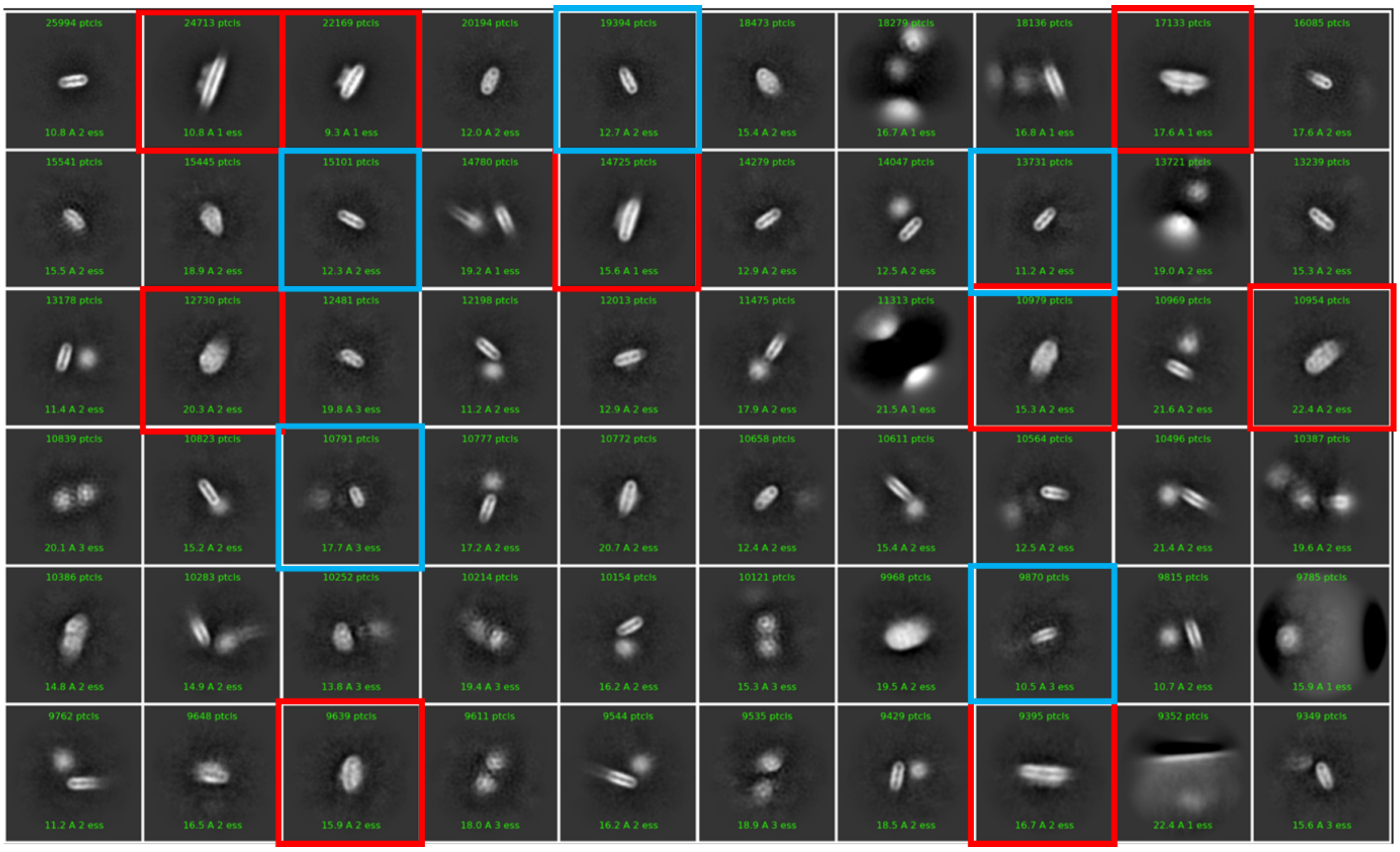

Supplementary Figure 3: Example of major 2D classes which were encountered during the cryo-electron microscopy workflow. Classes which were picked for further refinement and eventual 3D reconstruction of PSII complexes are highlighted with red boxes. A large number of the remaining classes appear to contain a small unknown particle. Some examples of these classes highlighted with a blue box. This small unknown particle is presumed to be isolated light-harvesting complexes, which dissociated from PS II following the high light treatment.

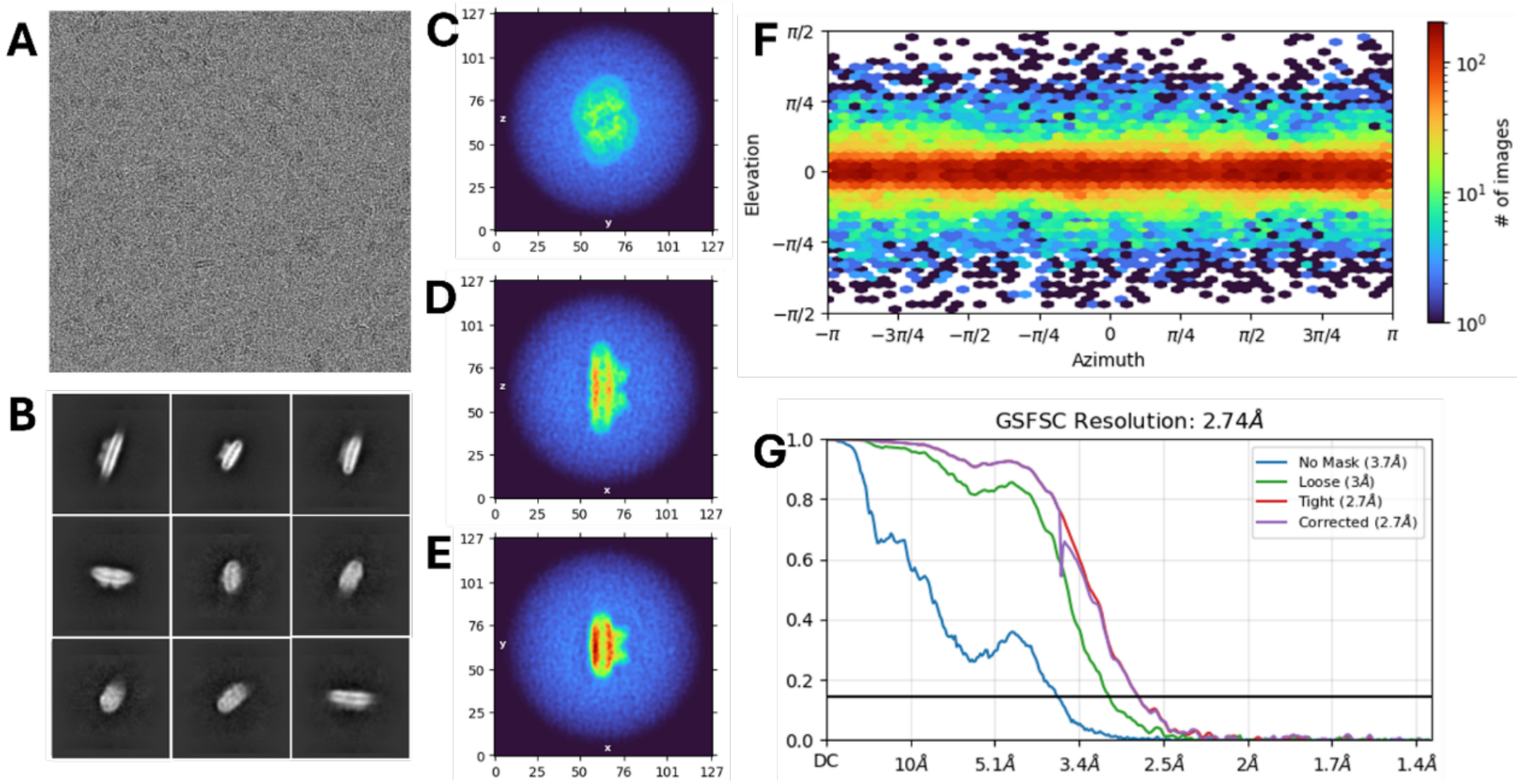

Supplementary Figure 4: Cryo-electron microscopy workflow. **(A)** Example of one of the 15,511 micrographs collected. Micrographs were collected at 165,000 X magnification. **(B)** Example of some of the 2D classes used to separate the 2,002,863 particles extracted from the micrographs into 57,560 "good particles". **(C-E)** Example of the best class from *ab initio* reconstruction. **(F)** Distribution of the orientation of the particles used to generate the best class from *ab initio* reconstruction. **(G)** Fourier Shell Correlation curves for the refined maps from the best classes of *ab initio* reconstruction **(C)**.

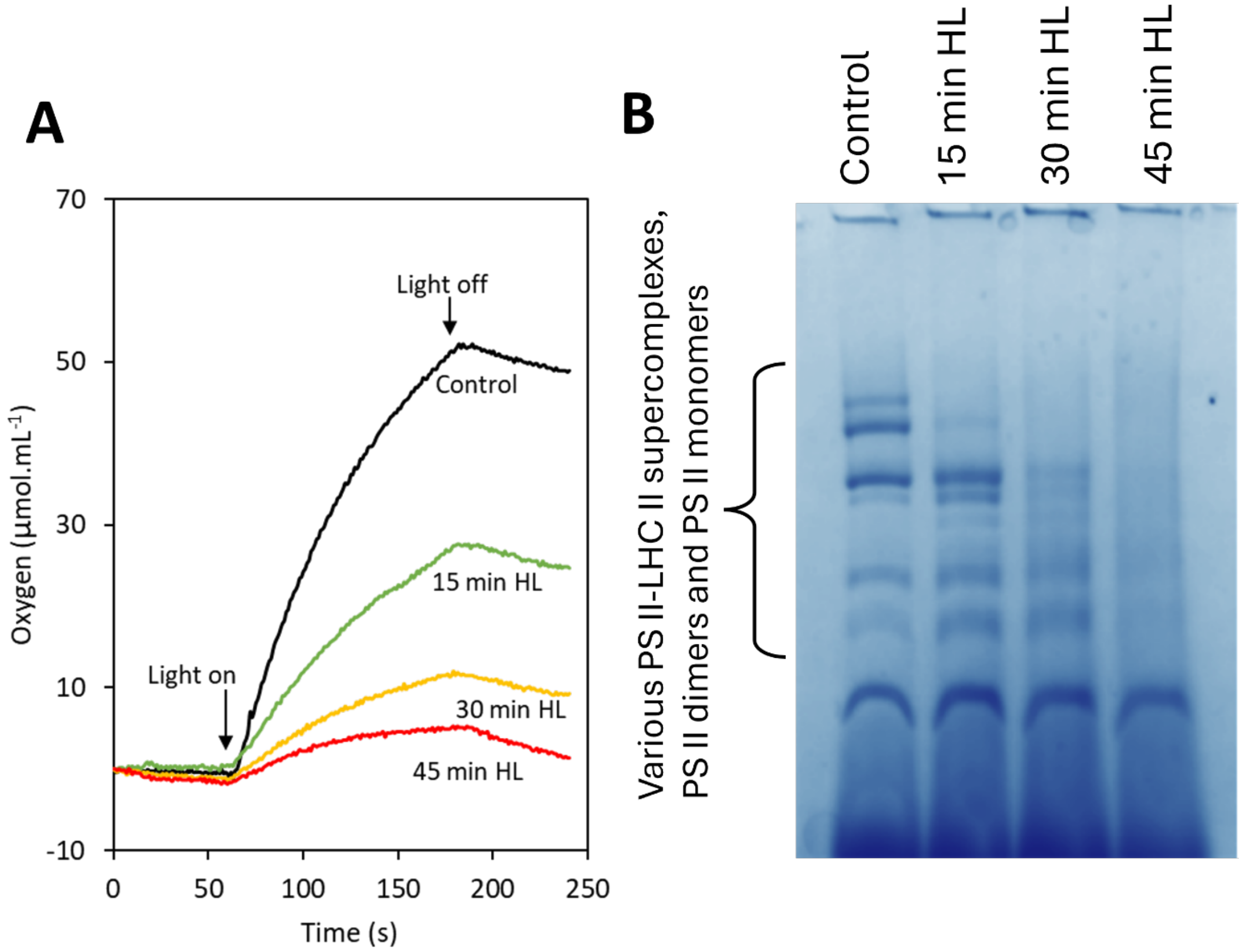

Supplementary Figure 5: The effect of high light (HL) on the O_2-evolution activity of photosystem II complexes of Arabidopsis. **(A)** Oxygen evolution measurements showing the rate of oxygen production in solubilised photosystem II after 0, 15, 30 and 45 mins of high light (2000 µE). Oxygen measurements were performed on solubilised photosystem II complexes isolated from sucrose gradients at a concentration of 10 µg/mL chlorophyll. PPBQ and FeCN were used as electron acceptors. The initial rates of oxygen evolution after 0,15, 30 and 45 mins of HL are 188, 99, 50 and 25 µmol O_2.mg $Chl^{-1}.h^{-1}$, respectively. **(B)** Blue-native gel showing the solubilised photosystem II complexes present in the sample after 0, 15, 30 or 45 mins of high light (2000 µE). In each lane PSII complexes containing 10 µg of Chl were loaded.

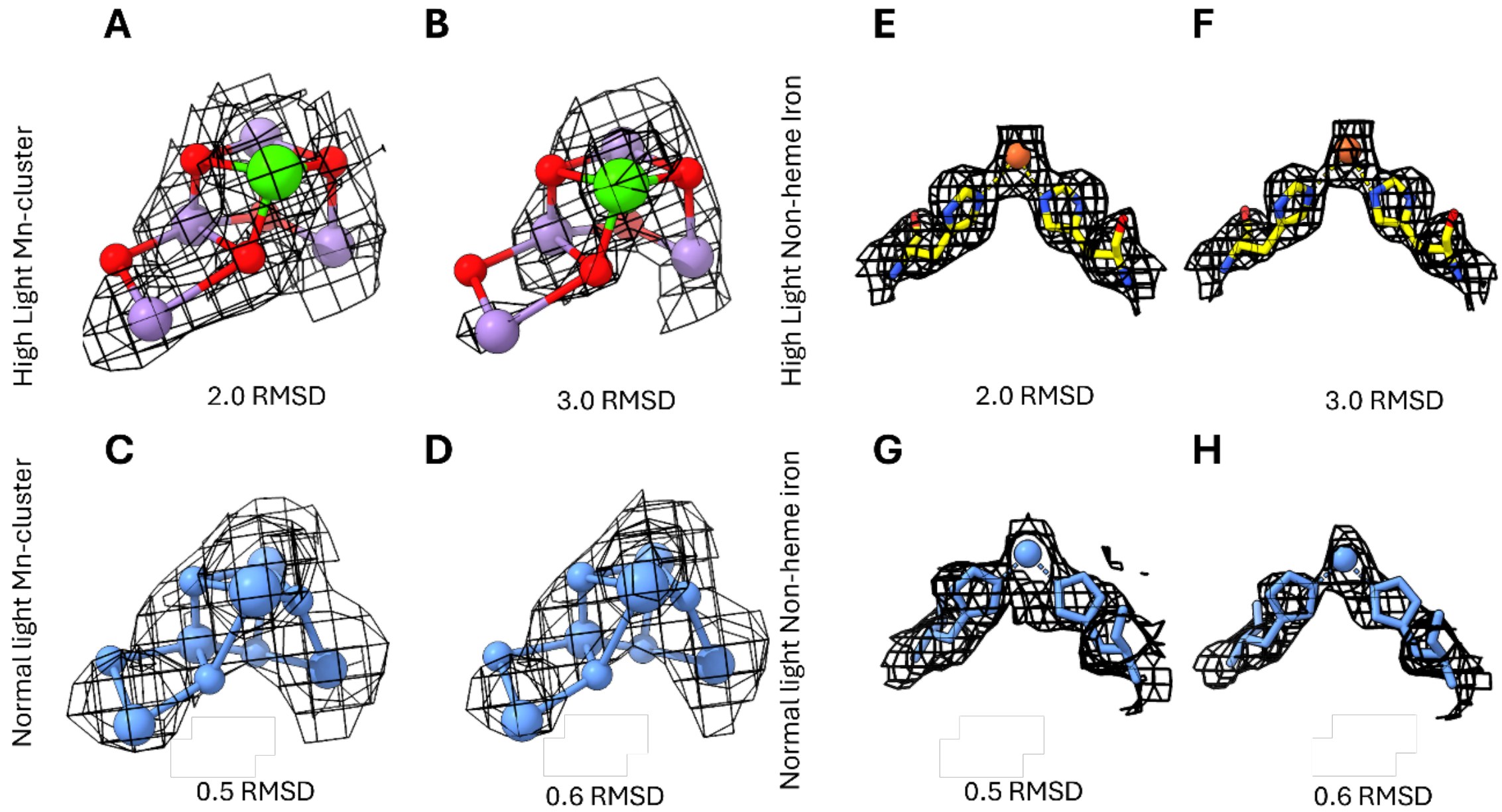

Supplementary Figure 6: Coulomb potential density around the Mn-cluster and non-heme iron (NHI) in the high-light and control structures. **(A,B)** Coulomb potential density within 2.0 Å of the Mn-cluster in the high light structure at a contour level of **(A)** 2.0 RMSD or **(B)** 3.0 RMSD. The Mn-cluster is shown as a collection of red, purple and green spheres. The Coulomb potential density from the high light map is shown as a black mesh. **(C,D)** Coulomb potential density within 2.0 Å of the Mn-cluster in the control structure (9I4T). The map used for the control structure has been low-pass filtered to 2.74 Å to match the global resolution of the high-light map. For this reprocessed map, contour levels of 0.5 RMSD **(C)** and 0.6 RMSD **(D)** are comparable to the 2.0 RMSD and 3.0 RMSD contour levels shown in panels **(A)** and **(B)**, respectively. The Mn-cluster in the control structure is shown as a collection of blue spheres. The Coulomb potential density from the reprocessed control map is shown as a black mesh. For comparison to the Mn-cluster, the Coulomb potential density is also shown within 2.0 Å around the NHI at the same contour levels **(E-H)**. The NHI (orange sphere) along with the ligating D1:His215 and D2:His214 residues are shown as yellow sticks in the high-light structure **(E-F)**. The NHI and histidine ligands are shown in blue in the control structure **(G-H)**.

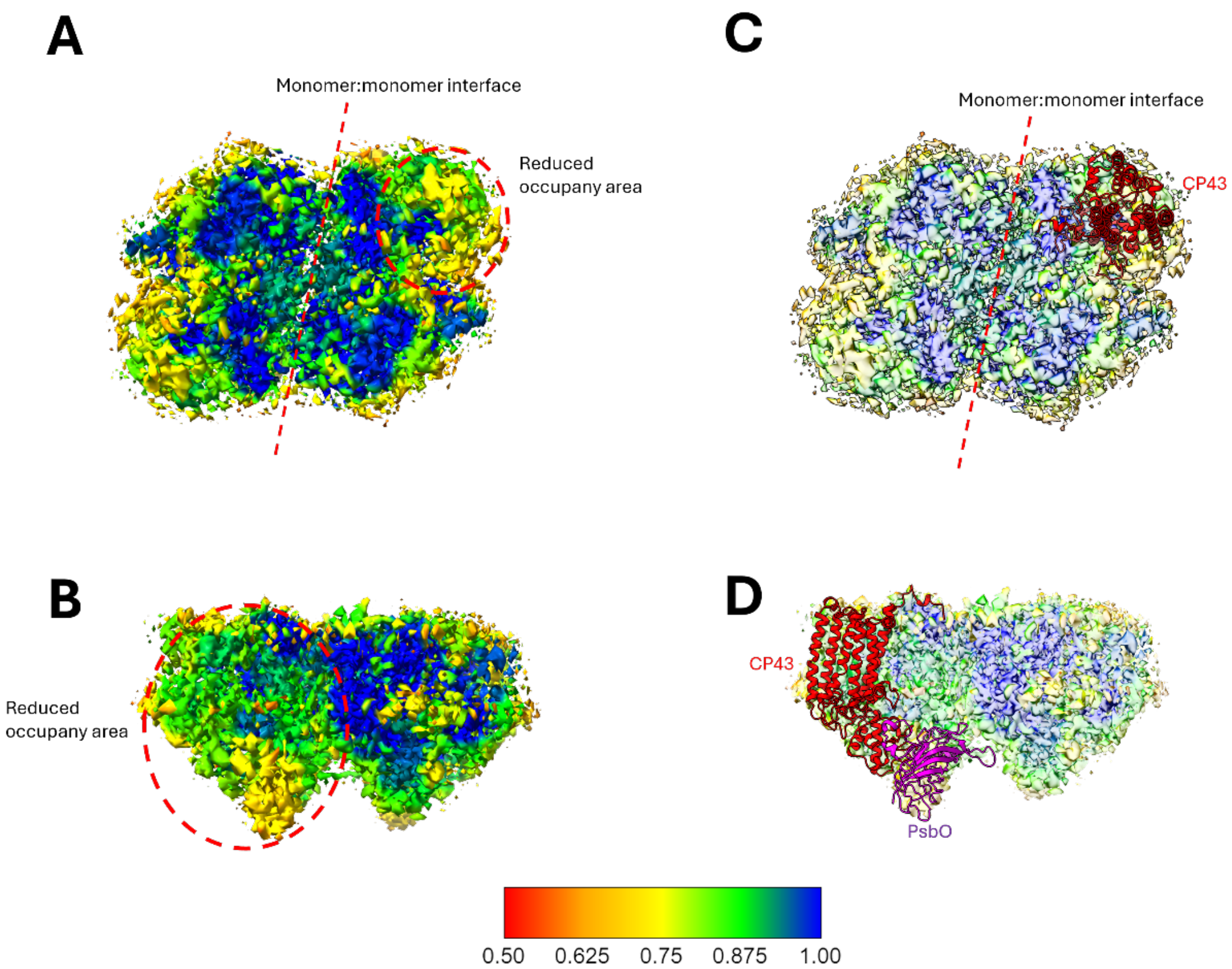

Supplementary Figure 7: Occupancy of the high-light Coulomb potential map was estimated using the software OccuPy. **(A)** Top view of the high-light Coulomb potential map, coloured to represent the relative occupancy. The colour key is shown at the bottom of the figure. A red line indicates the monomer:monomer interface where both sides have C2 symmetry. On one monomer the low occupancy area is circled with a red dashed line. **(B)** Side view of the high-light Coulomb potential map, coloured to represent the relative occupancy. On one monomer the low occupancy area is circled with a red dashed line. **(C)** and **(D)** show the same maps from **(A)** and **(B)**, but with the CP43 and PsbO subunits modelled into the low occupancy areas of the map. CP43 is shown in red, PsbO is shown in pink.

‘

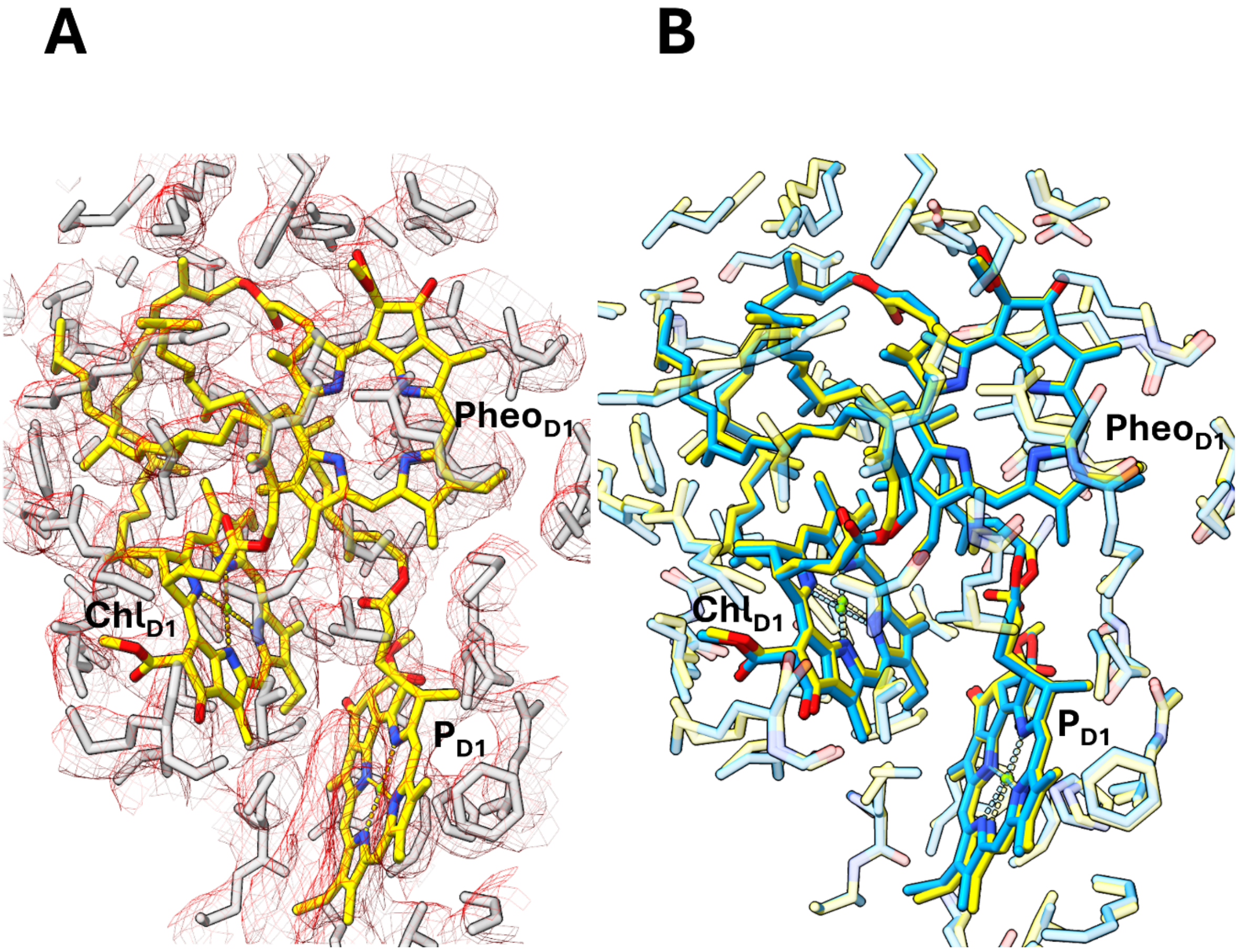

Supplementary Figure 8: (A) Fitting of the D1 chlorophyll molecules in the P680 region into the high-light Coulomb potential map. The pheophytin (Pheo) and chlorophyll molecules (Chl$_{D1}$ and P$_{D1}$) are shown in yellow, with red and dark blue sections indicating oxygen and nitrogen atoms, respectively. Residues within 5 Å of these cofactors are shown in grey. The high-light Coulomb potential map is shown around the selected residues in as a red mesh. **(B)** Comparison of the P680 region in the control structure (blue sticks) and the high-light structure (yellow sticks). The chlorophyll and pheophytin residues are opaque. Residues within 5 Å of these cofactors are shown as partially transparent.

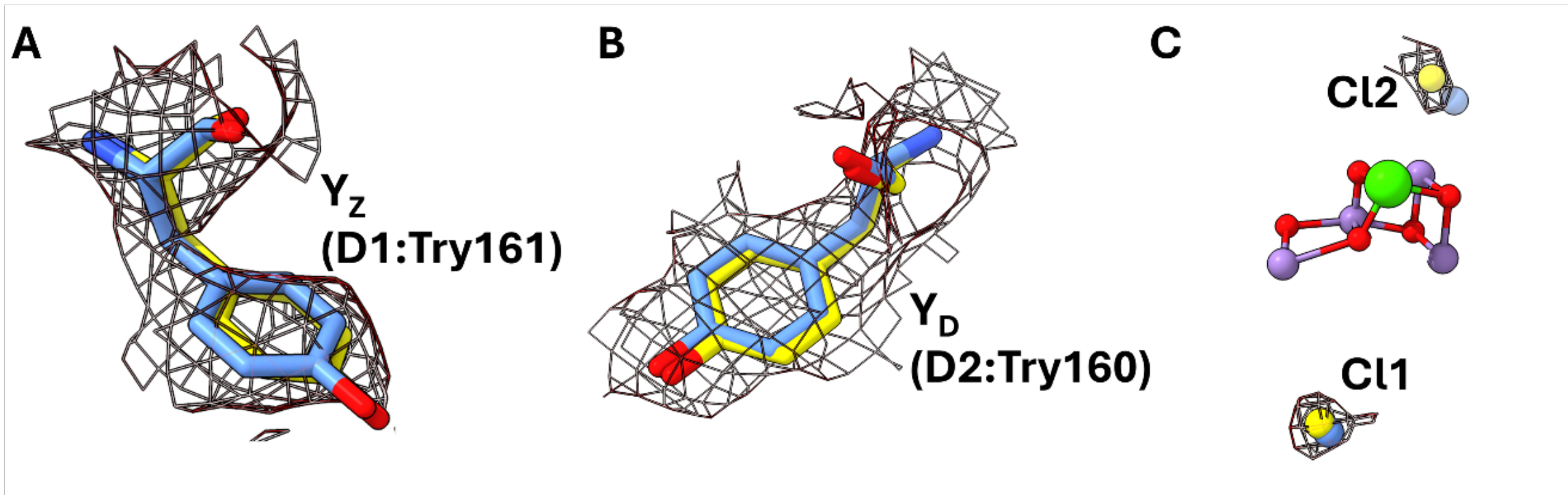

Supplementary Figure 9: Comparison of Y_z **(A)**, Y_D **(B)** and the Cl1 and Cl2 chloride ions **(C)** in in the control (blue sticks or spheres) and high-light (yellow sticks or spheres) structures. Oxygen and nitrogen atoms are represented by blue and red sections, respectively. In panel **(C)**, the Mn-cluster is also shown to help orientation. The high-light Coulomb potential map (2.74 Å) is shown at a contour level of 2.0 RMSD within 2.0 Å of the selected residues as a black mesh.

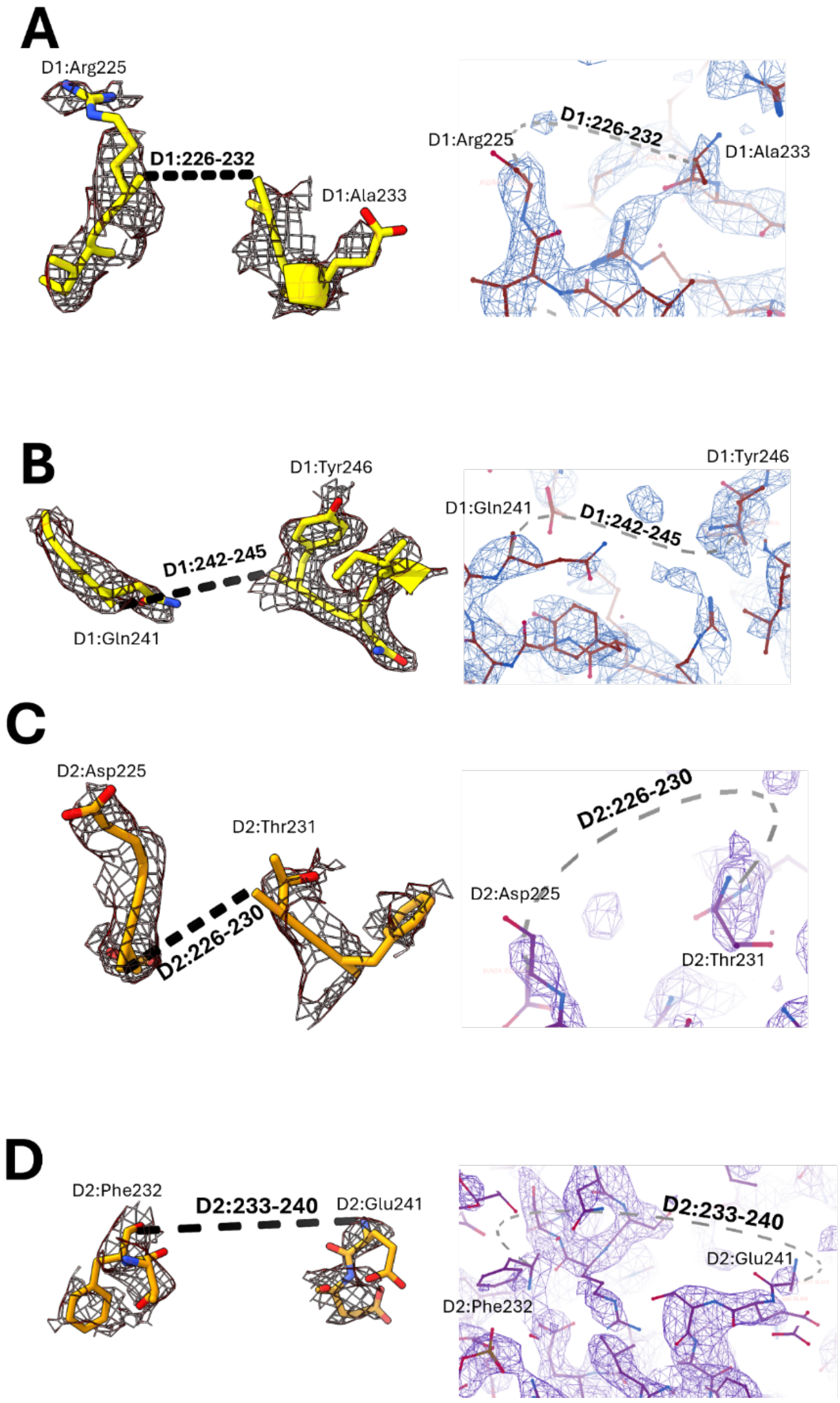

Supplementary Figure 10: The loss of electron density in the DE loops of the D1 and D2 proteins. Unmodelled sections are D1:226-232 **(A)**, D1:242-245 **(B)**, D2:226-230 **(C)**, D1:233-240 **(D)**. Each of the four unmodelled sections are shown using both a ChimeraX graphic (left) and a COOT graphic (right). This is because the structure can be seen more clearly in ChimeraX, but the electron density around the unmodelled section can be seen better in COOT. For the ChimeraX graphics D1 sections D1:225-231 and D1:241-246 are shown using yellow sticks. D2 sections D1:226-230 and D1:233-240 are shown using orange sticks. For all graphics red and blue sections represent oxygen and nitrogen atoms, respectively. The unmodelled residues are shown as a thick dashed line. Residues on each end of the unmodelled section are labelled. The high-light Coulomb potential map (2.74 Å) is shown at a contour level of 2.0 RMSD within 2.0 Å of the selected residues as a red mesh. For the COOT graphics residues are shown as red sticks for both D1 and D2. Coulomb potential density from the high-light map at a contour level of 2.06 RMSD is shown as a blue mesh. The unmodelled residues are shown as a dashed grey line. Residues on each end of the unmodelled section are labelled.

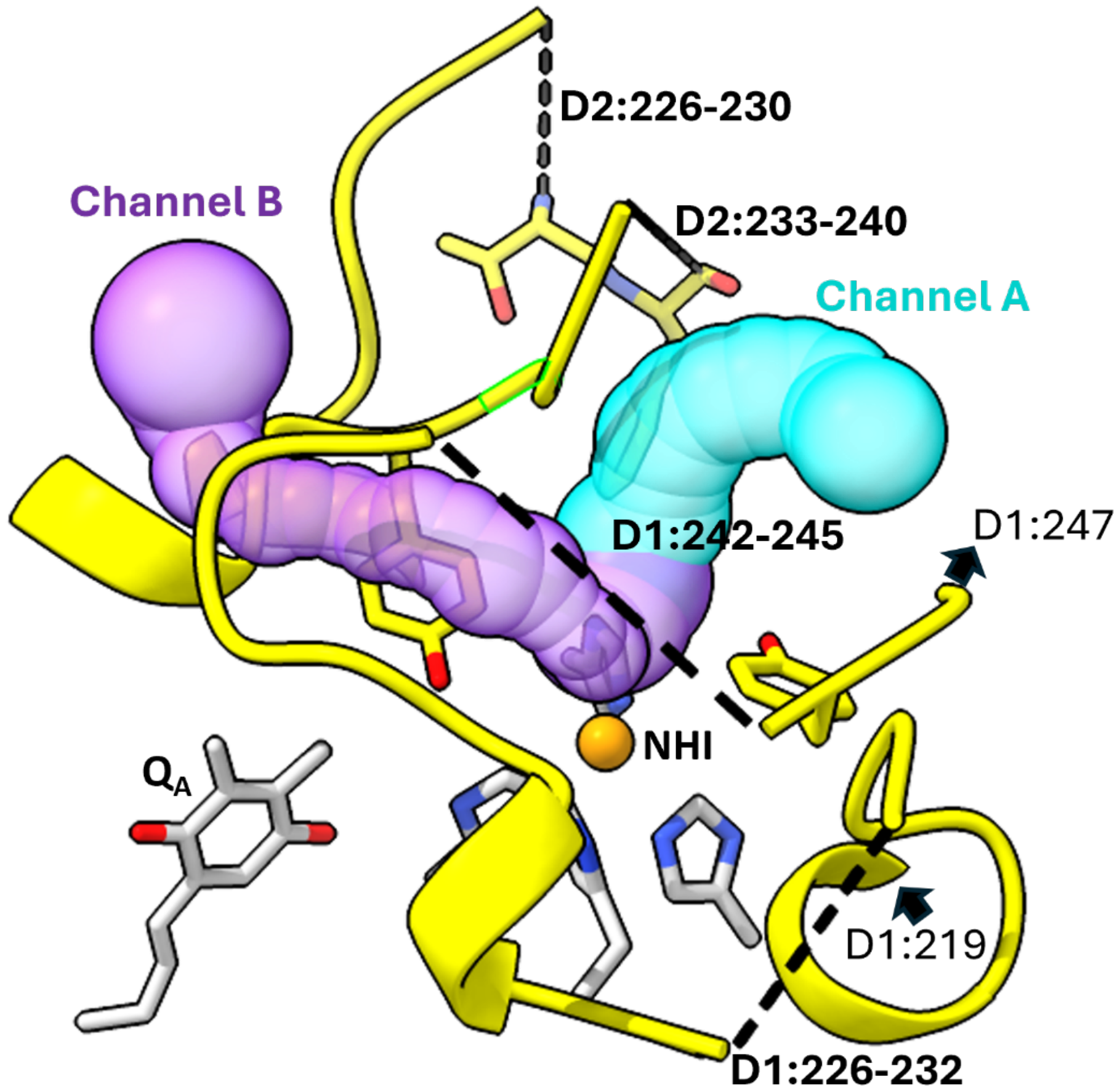

Supplementary Figure 11: The location of the unmodelled loops in relation to the acceptor side channels. D1 residues 219-247 and D2 residues 219-247 are shown as yellow ribbons, with red or blue sections indicating oxygen or nitrogen, respectively. Channel A is shown in cyan, and Channel B is shown in purple. Most of the side chains are not shown for clarity. Unmodelled loop regions are indicated by dashed black lines. Q_A, and the histidine ligands to the non-heme iron are shown as white sticks, the non-heme iron (NHI) is shown as an orange sphere.

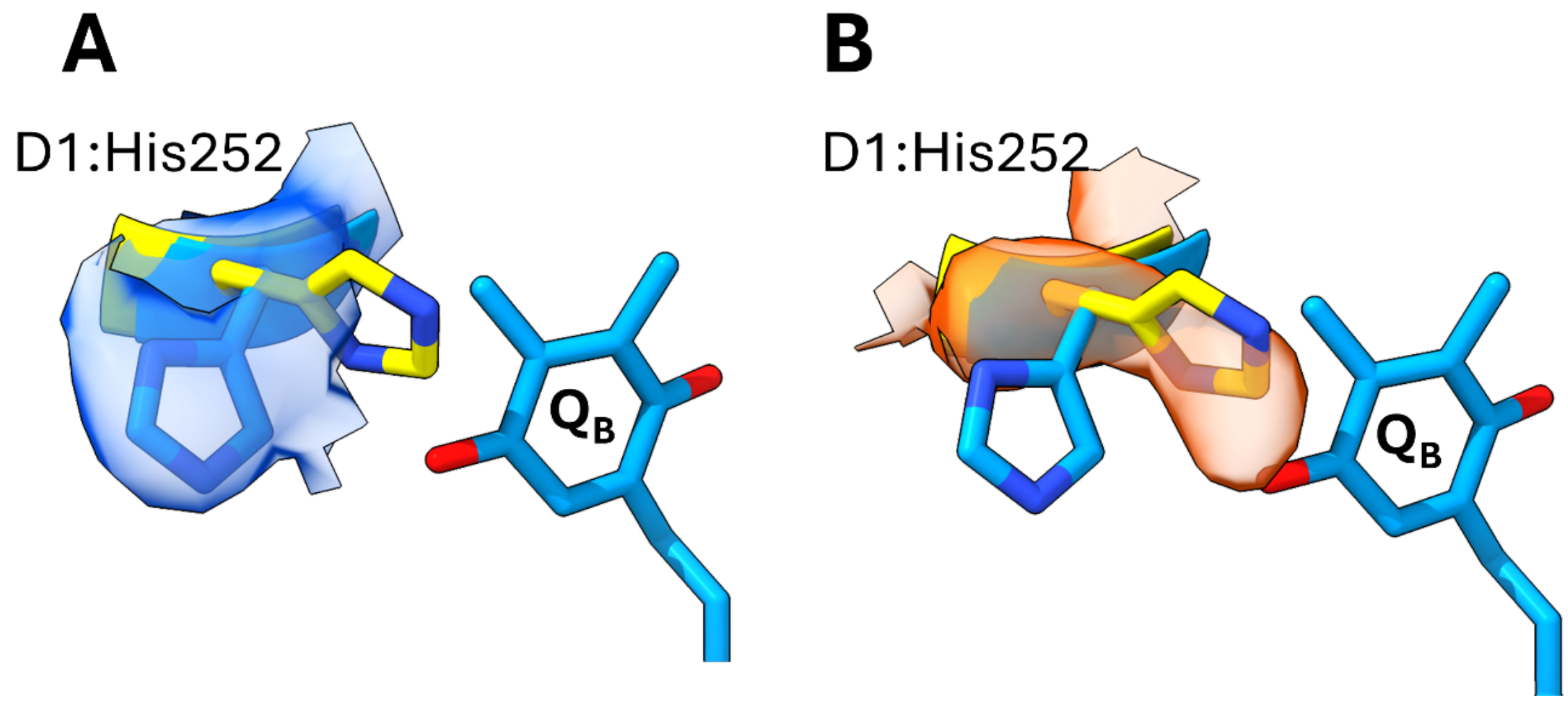

Supplementary Figure 12: The difference between the D1:His252 residue side chain orientation in the control and high light structures. The D1:His252 residues is shown for both the control (blue sticks) and the high-light model (yellow sticks). The Q_B cofactor from the control is also shown to help viewer orientation. The Coulomb potential map from the control is shown as a blue surface **(A)** and that of the high-light particles is shown as a red surface **(B).**

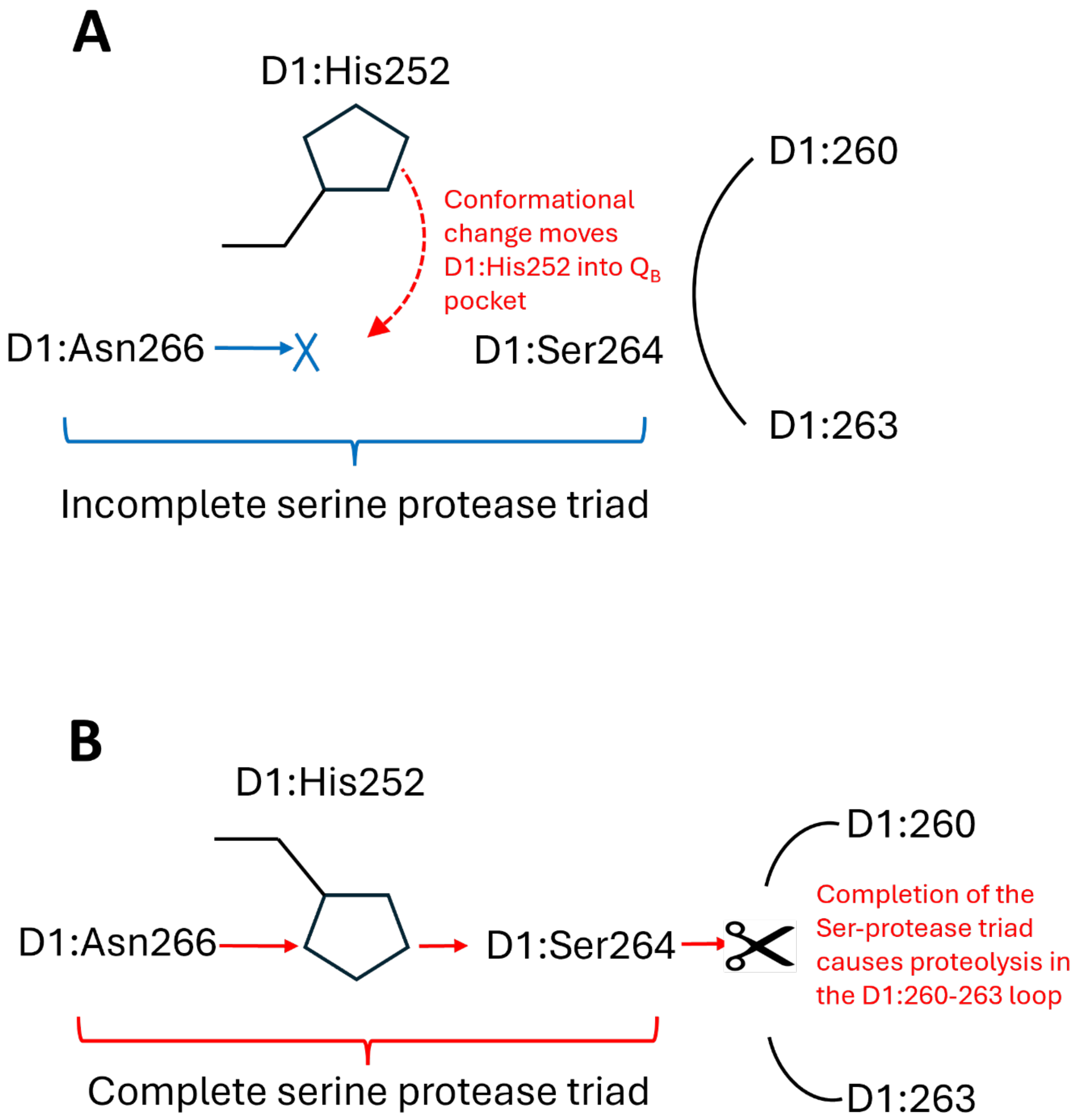

Supplementary Figure 13: Schematic view of the hypothesized arrangement of D1:Asn266, D1:His252 and D1:Ser264 **(A)** before and **(B)** after the high-light treatment. A red arrow indicates the direction of movement of the D1:His252 residue following high-light treatment. The scissors icon indicates that the Serine-protease triad is able to cut the D1 protein between the D1:260 and D1:264 residues.

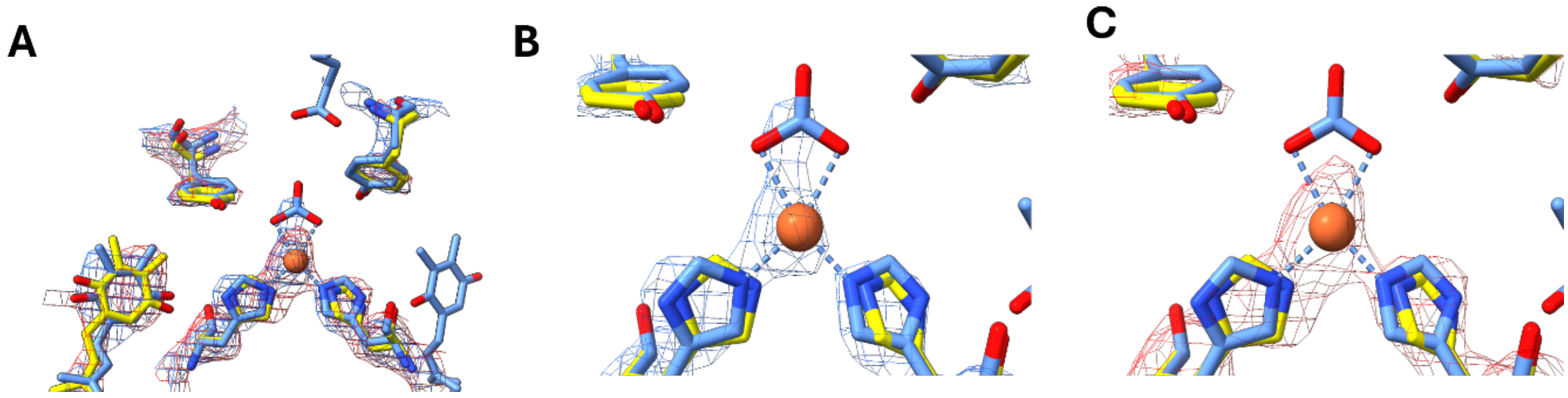

Supplementary Figure 14: (A) Comparison of the bicarbonate environment in the control (blue sticks) and high-light (yellow sticks) structures. Oxygen and nitrogen atoms are represented by blue and red sections, respectively, the non-heme iron is shown as an orange sphere. The control Coulomb potential map, reprocessed to 2.74 Å resolution, is shown as a blue mesh. The high-light Coulomb potential map (2.74 Å) is shown as a red mesh. Both potential maps are shown at comparable contour levels. **(B)** The fitting of the bicarbonate as a bidentate ligand into the re-processed control Coulomb potential map at a contour level of 0.6 RMSD. **(C)** Absence of a Coulomb potential signal for bicarbonate at a contour level of 3 RMSD.

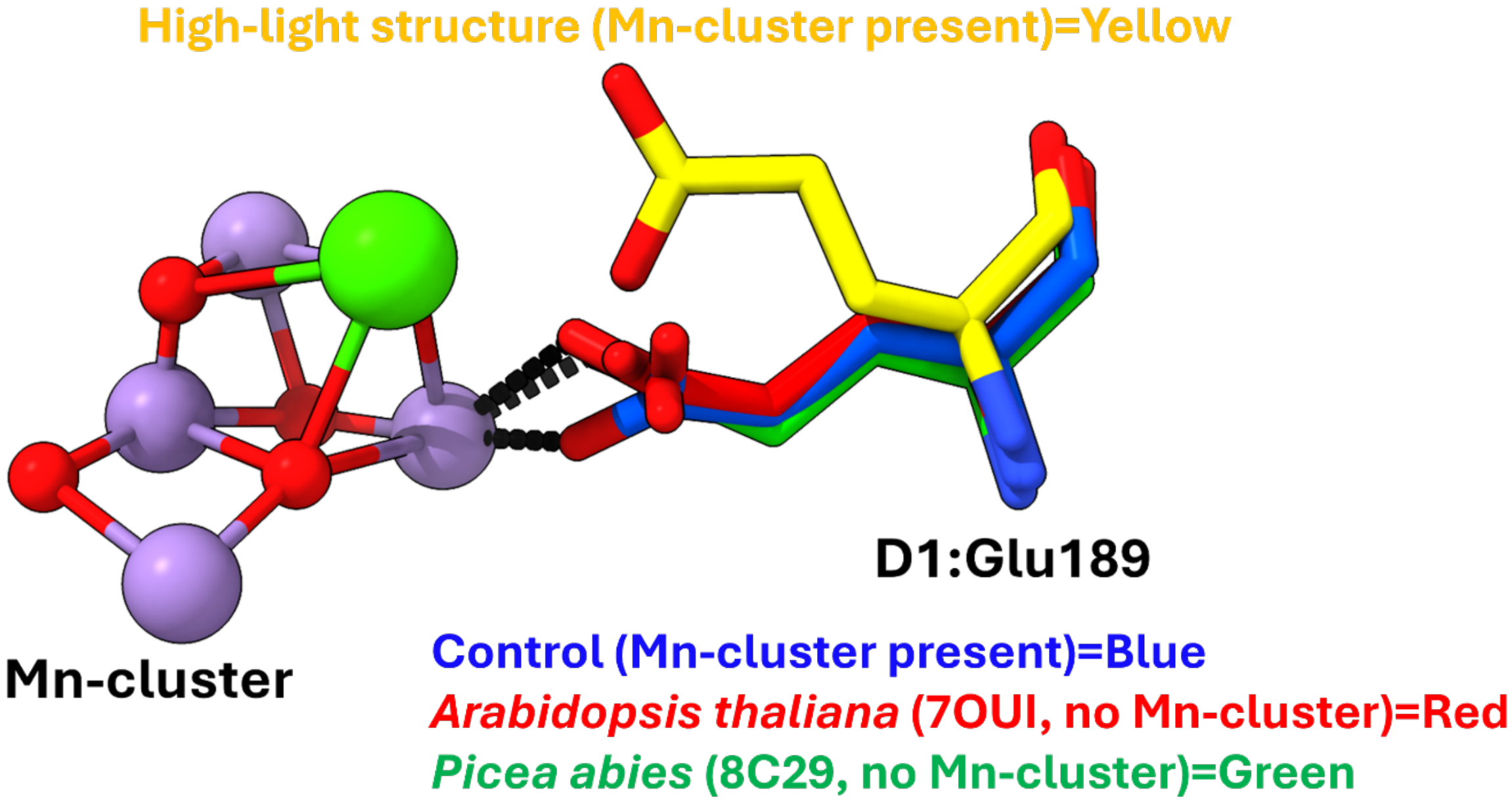

Supplementary Figure 15: Position of the D1:Glu189 residue relative to the Mn-cluster in *Arabidopsis thaliana* control **(blue)** (9I4T, Forsman et al. 2026), *Arabidopsis thaliana* without PsbP and PsbQ **(red)** (7OUI, Graca et al., 2021)**,** *Picea abies* **(green)** (8C29, Opatikova et al., 2023) and *Arabidopsis thaliana* high-light **(yellow)** (present structure). The Mn-cluster is shown as a collection of red, purple and green spheres to represent oxygen, manganese and calcium ions, respectively. On the D1:Glu189 residues, red and blue sections represent oxygen and nitrogen atoms, respectively.

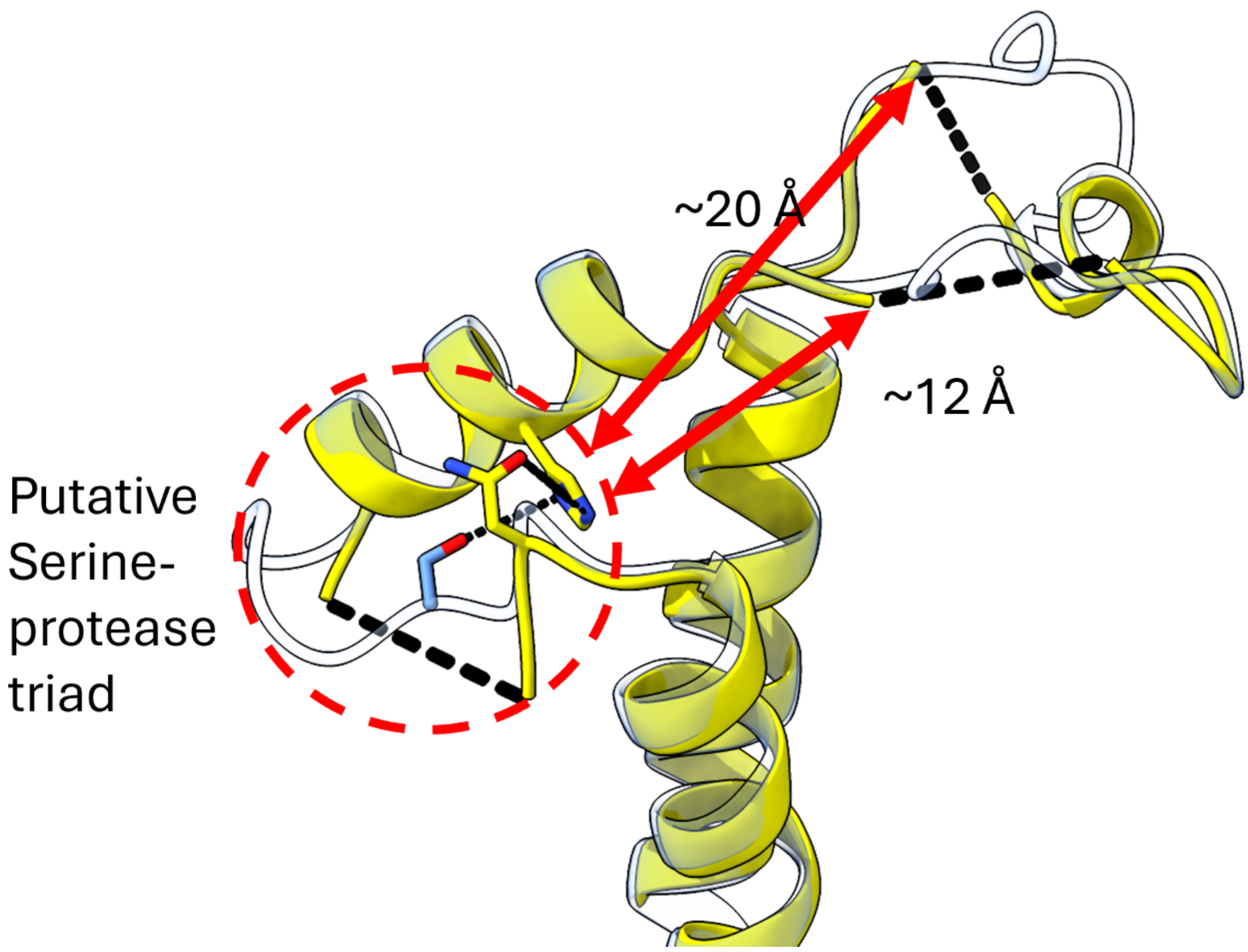

Supplementary Figure 16: Approximate distances from the putative serine-protease at the Q_B-binding site, to the unmodelled sections of the D1 DE loop. The putative serine-protease is circled with a red circle. The approximate distance from the serine-protease to the closest point in the unmodelled D1:226-232 section is 23 Å. The approximate distance from the serine-protease to the closest point in the unmodelled D1:242-245 section is 14 Å. The location of the loops in the control structure are shown as transparent white ribbons.

Supplementary Table 1: Statistics of cryo-EM data and structural analysis of the high-light Photosystem II structure refined at 2.74 Å resolution.

**Data collection and processing**	
Hardware	
Microscope	Titan Krios
Detector (mode)	FEI Falcon IV
Voltage (keV)	300
Spherical aberration	2.7
Magnification	165,000 x
Electron exposure (e/Å^2)	40
Defocus range (µm)	-0.8 to -2.0 (0.2 increments)
Pixel size (Å)	0.7155
Symmetry imposed	C2
Initial particle images (no.)	2,002,863
Final particle images (no.)	57,560
Map resolution (Å)	2.74
FSC threshold	0.143
**Refinement**	
Initial models used (PDB code)	9I4T
Model composition	
Non-hydrogen atoms	39298
Protein residues	4174
Ligands	120
Water molecules	52
B factors (Å^2)	
Protein (mean)	52.91
Ligand (mean)	50.23
R.m.s. deviations	
Bond lengths (Å)	0.009
Bond angles (°)	1.009
Validation	
MolProbity score	2.55
Clash score	33.26
Rotamer outliers (%)	2.80
Ramachandran plot	
Favored (%)	96.73
Allowed (%)	3.20
Outliers (%)	0.07

Supplemental Table 2: Details of the individual Photosystem II subunits. The protein name is shown in bold, while the gene name is contained within parenthesis. The accession code used to identify the protein in UniProt is shown before the sequence. The sequences have amino acids letters colored in red, blue or black: **black** letters, all the amino acids that were modelled; **blue** amino acids are modelled in the control structure, but not the high-light structure; **red**, amino acids neither modelled in the control nor in the high-light structure of *Arabidopsis thaliana*. Each subunit is shown fit into the locally sharpened electron density map at a contour level of 2.3 RMSD. Each subunit from the model is shown with red sticks. The electron density map surrounding the subunit within a distance of 2 Å is shown as a blue surface. The location of the specific subunit within the PS II dimer is shown in red, with the rest of the dimer shown in white.

Chain ID	Protein Name (Gene name)	MW (kDa)	Sequence (mature protein)	Model fit into the electrostatic potential map	Location of subunit within the PS II dimer
A,a	**D1** (PsbA)	37.98	>P83755<br>MTAILERRESESLWGRFCNWITSTENRLYI<br>GWFGVLMIPTLLTATSVFIIAFIAAPPVDIDG<br>IREPVSGSLLYGNNIISGAIIPTSAAIGLHFY<br>PIWEAASVDEWLYNGGPYELIVLHFLLGVA<br>CYMGREWELSFRLGMRPWIAVAYSAPVA<br>AATAVFLIYPIGQGSFSDGMPLGISGTFNF<br>MIVFQAEHNILMHPFHMLGVAGVFGGSLF<br>SAMHGSLVTSSLIRETTENESANEGYRFG<br>QEEETYNIVAAHGYFGRLIFQYASFNNSRS<br>LHFFLAAWPVVGIWFTALGISTMAFNLNGF<br>NFNQSVVDSQGRVINTWADIINRANLGME<br>VMHERNAHNFPLDLAA	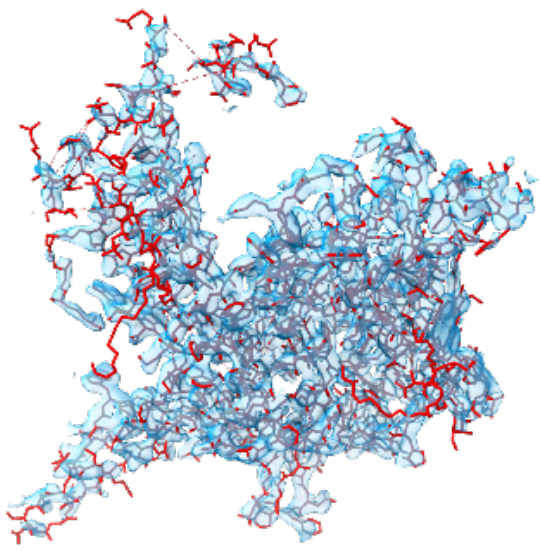	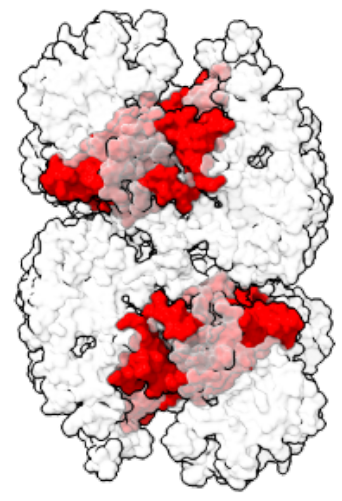
B,b	**CP47** (PsbB)	56.04	>P56777<br>MGLPWYRVHTVVLNDPGRLLAVHIMHTAL<br>VAGWAGSMALYELAVFDPSDPVLDPMWR<br>QGMFVIPFMTRLGITNSWGGWSITGGTIT<br>NPGIWSYEGVAGAHIVFSGLCFLAAIWHW<br>VYWDLEIFSDERTGKPSLDLPKIFGIHLFLS<br>GVACFGFGAFHVTGLYGPGIWVSDPYGLT<br>GKVQPVNPAWGVEGFDPFVPGGIASHHIA<br>AGTLGILAGLFHLSVRPPQRLYKGLRMGNI<br>ETVLSSSIAAVFFAAFVVAGTMWYGSATT<br>PIELFGPTRYQWDQGYFQQEIYRRVSAGL<br>AENQSLSEAWAKIPEKLAFYDYIGNNPAK<br>GGLFRAGSMDNGDGIAVGWLGHPVFRNK<br>EGRELFVRRMPTFFETFPVVLVDGDGIVR<br>ADVPFRRAESKYSVEQVGVTVEFYGGEL<br>NGVSYSDPATVKKYARRAQLGEIFELDRA<br>TLKSDGVFRSSPRGWFTFGHASFALLFFF<br>GHIWHGARTLFRDVFAGIDPDLDAQVEFG<br>AFQKLGDPTTKRQAV	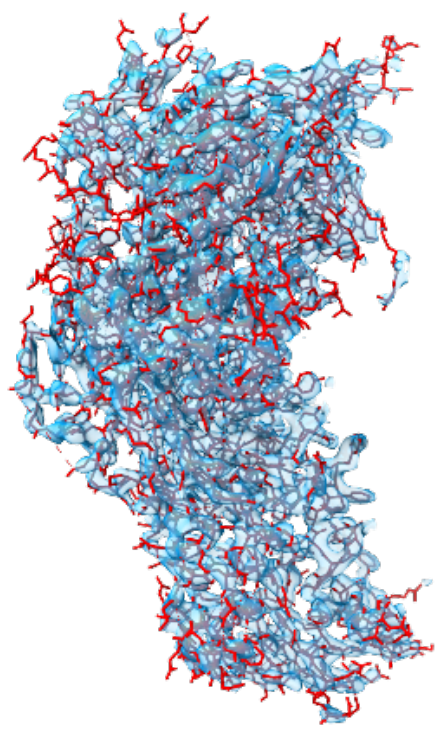	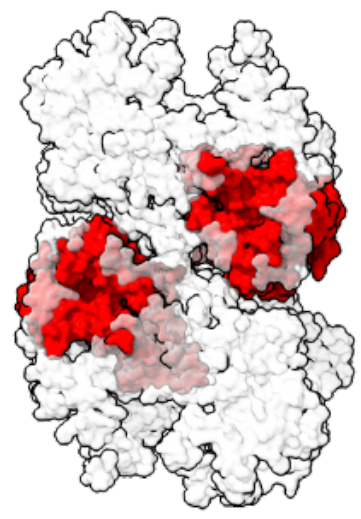
C,c	**CP43** (PsbC)	50.04	>P56778<br>TLFNGTLALAGRDQETTGFAWWAGNARLI<br>NLSGKLLGAHVAHAGLIVFWAGAMNLFEV<br>AHFVPEKPMYEQGLILLPHLATLGWGVGP<br>GGEVIDTFPYFVSGVLHLISSAVLGFGGIY<br>HALLGPETLEESFPFFGYVWKDRNKMTTI<br>LGIHLILLGVGAFLLVFKALYFGGVYDTWA<br>PGGGDVRKITNLTLSPSIIFGYLLKSPFGGE<br>GWIVSVDDLEDIIGGHVWIGSICIFGGIWHI<br>LTKPFAWARRALVWSGEAYLSYSLAALSV<br>CGFIACCFVWFNNTAYPSEFYGPTGPEAS<br>QAQAFTFLVRDQRLGANVGSAQGPTGLG<br>KYLMRSPTGEVIFGGETMRFWDLRAPWL<br>EPLRGPNGLDLSRLKKDIQPWQERRSAEY<br>MTHAPLGSLNSVGGVATEINAVNYVSPRS<br>WLSTSHFVLGFFLFVGHLWHAGRARAAA<br>AGFEKGIDRDFEPVLSMTPLN	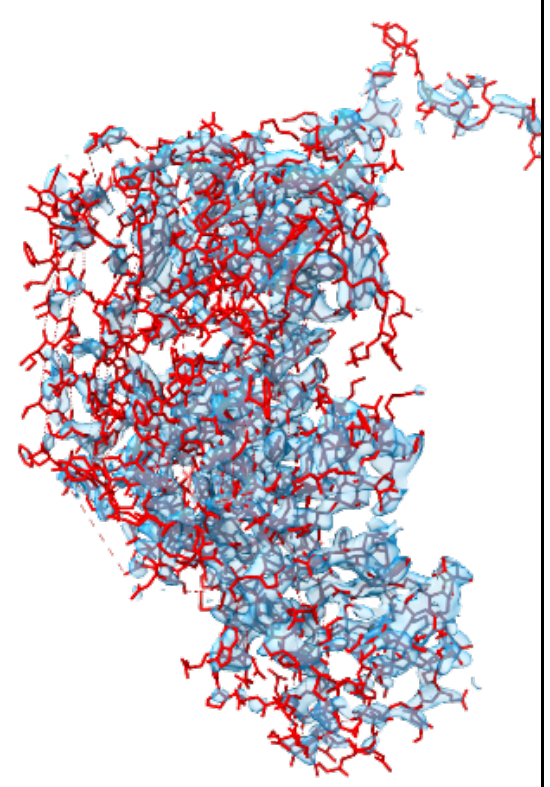	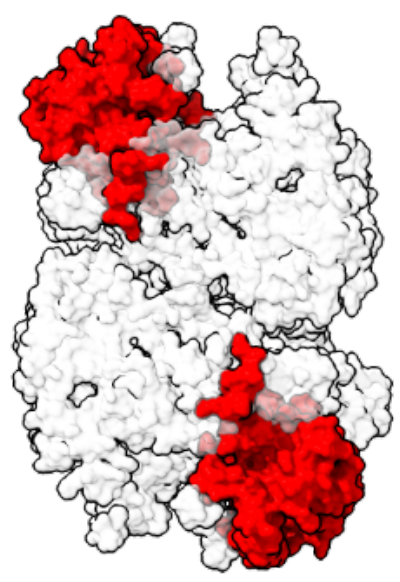

D,d	**D2**<br>(PsbD)	39.42	>P56761<br>TIALGKFTKDEKDLFDIMDDWLRRDRFVFV<br>GWSGLLLFPCAYFALGGWFTGTTFVTSW<br>YTHGLASSYLEGCNFLTAAVSTPANSLAH<br>SLLLLWGPEAQGDFTRWCQLGGLWAFVA<br>LHGAFALIGFMLRQFELARSVQLRPYNAIA<br>FSGPIAVFVSVFLIYPLGQSGWFFAPSFGV<br>AAIFRFILFFQGFHNWTLNPFHMMGVAGV<br>LGAALLCAIHGATVENTLFEDGDGANTFR<br>AFNPTQAEETYSMVTANRFWSQIFGVAFS<br>NKRWLHFFMLFVPVTGLWMSALGVVGLA<br>LNLRAYDFVSQEIRAAEDPEFETFYTKNILL<br>NEGIRAWMAAQDQPHENLIFPEEVLPRGN<br>AL	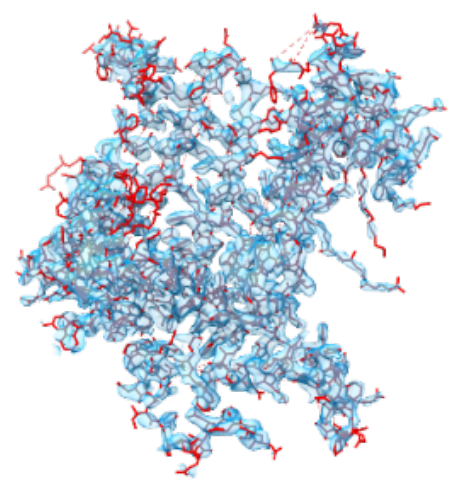	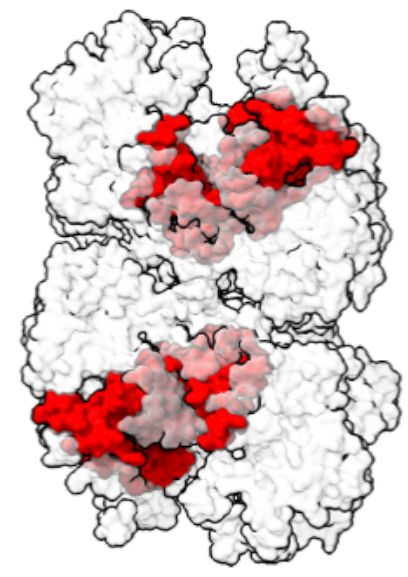
E,e	**Cytochrome $b_{559}\alpha$**<br>(PsbE)	9.39	>P56779<br>MSGSTGERSFADIITSIRYWVIHSITIPSLFI<br>AGWLFVSTGLAYDVFGSPRPNEYFTESR<br>QGIPLITGRFDSLEQLDEFSRSF	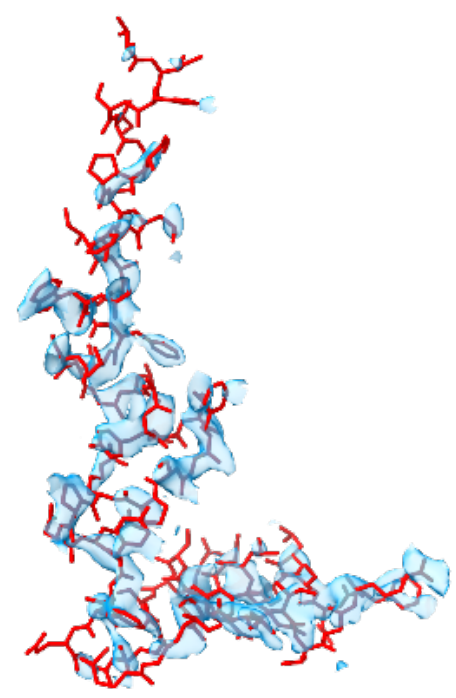	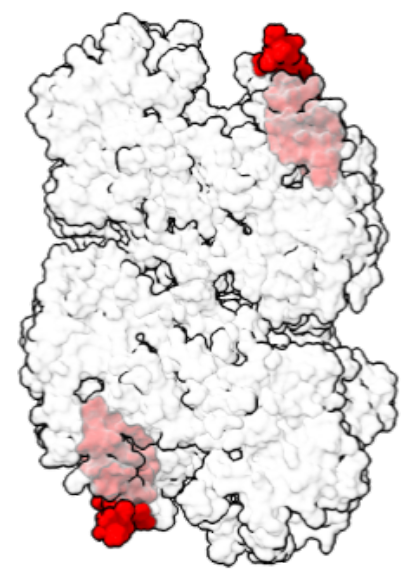
F,f	**Cytochrome $b_{559}\beta$**<br>(PsbF)	4.42	>P62095<br>MTIDRTYPIFTVRWLAVHGLAVPTVFFLGSI<br>SAMQFIQR	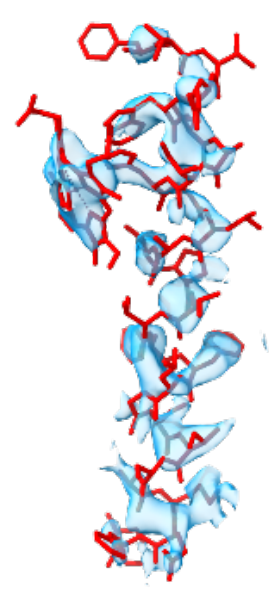	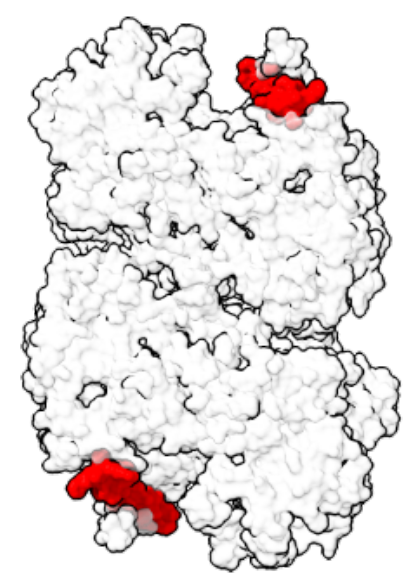
H,h	**Photosystem II reaction center protein H**<br>(PsbH)	7.57	>P56780<br>ATQTVEDSSRSGPKPTTVGALLKPLNSEY<br>GKVAPGWGTTPLMGVAMALFAVFLSIILEI<br>YNSSVLLDGISVN	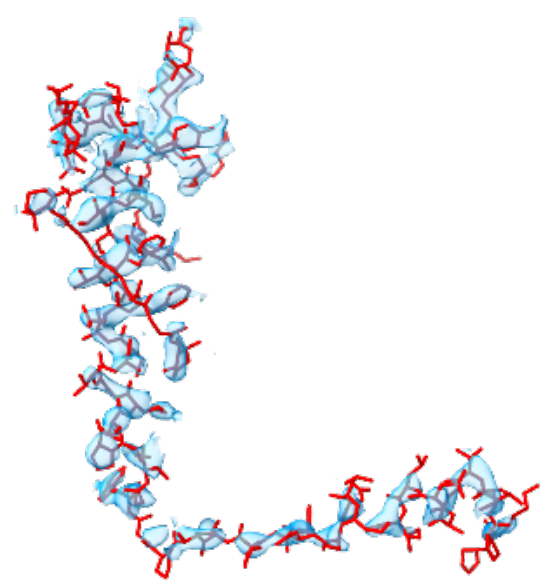	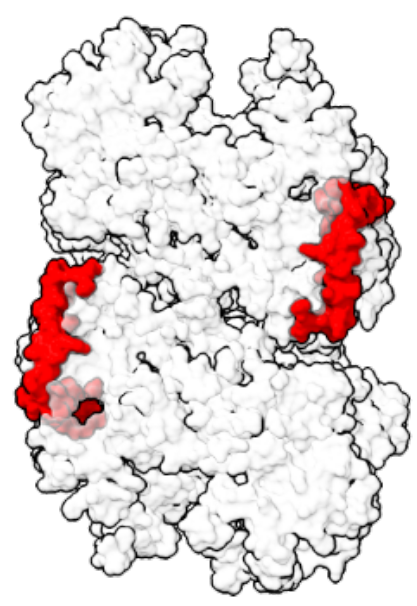

I,i	**Photosy stem II reaction center protein I** (PsbI)	4.17	>P62100<br>MLTLKLFVYTVVIFFVSLFIFGFLSNDPGRN PGREE	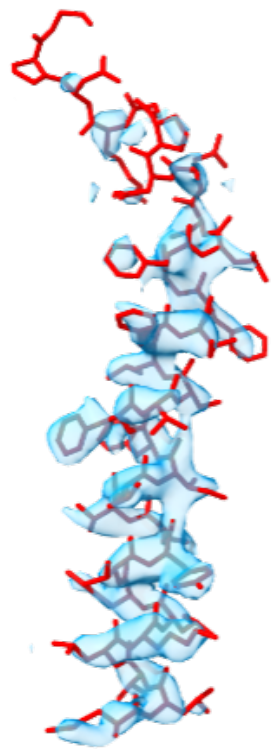	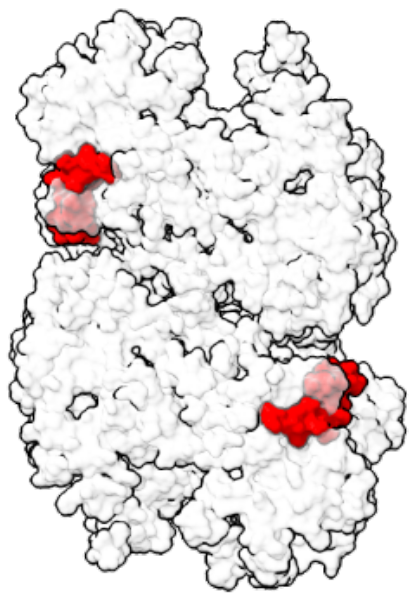
K,k	**Photosy stem II reaction center protein K** (PsbK)	4.24	>P56782<br>KLPEAYAFLSPIVDVMPVIPLFFLLLAFVWQ AAVSFR	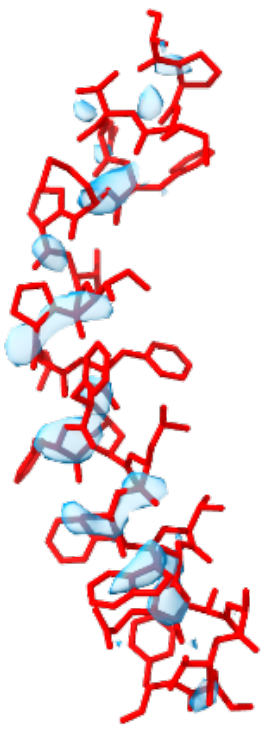	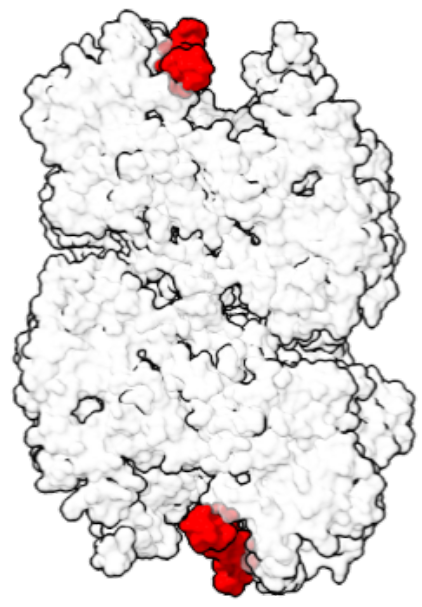
L,l	**Photosy stem II reaction center protein L** (PsbL)	4.47	>P60129<br>MTQSNPNEQSVELNRTSLYWGLLLIFVLA VLFSNYFFN	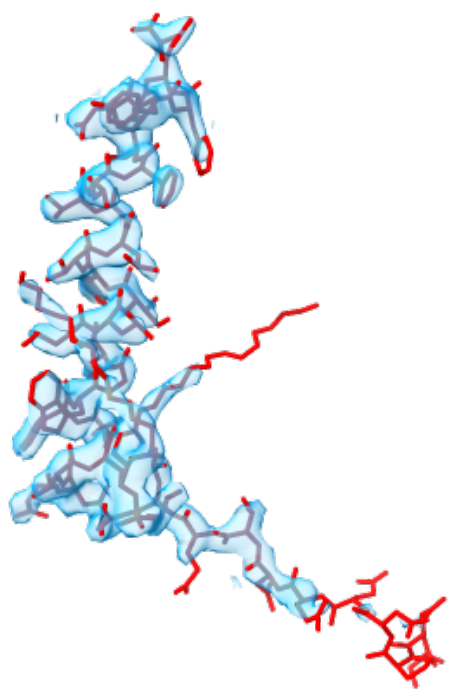	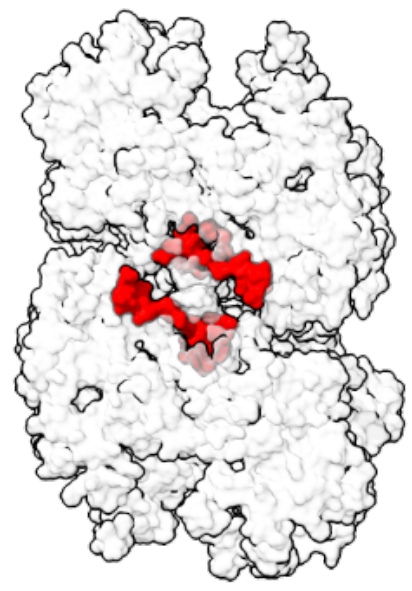
M,m	**Photosy stem II reaction center protein M** (PsbM)	3.78	>P62109<br>MEVNILAFIATALFILVPTAFLLIIYVKTVSQN D	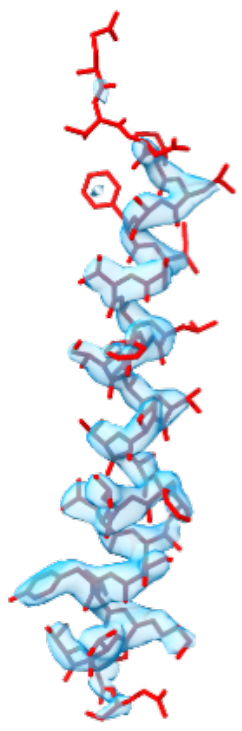	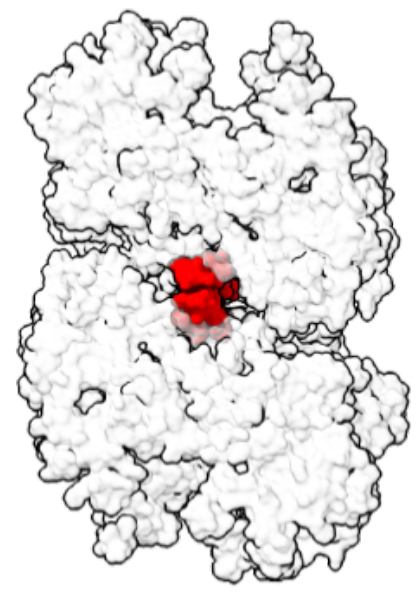

O,o	**Oxygen-evolving enhancer protein 1-1** (PsbO1)	26.57	>P23321<br>EGAPKRLTYDEIQSKTYMEVKGTGTANQC<br>PTVDGGSETSFKPGKYAGKKFCFEPTSFT<br>VKADSVSKNAPPEFQNTKLMTRLTYTLDEI<br>EGPFEVSSDGSVNFKEEDGIDYAAVTVQL<br>PGGERVPFLFTVKQLDASGKPDSFTGKFL<br>VPSYRGSSFLDPKGRGGSTGYDNAVALP<br>AGGRGDEEELVKENVKNTAASVGEITLKV<br>TKSKPETGEVIGVFESLQPSDTDLGAKVP<br>KDVKIQGVWYGQLE	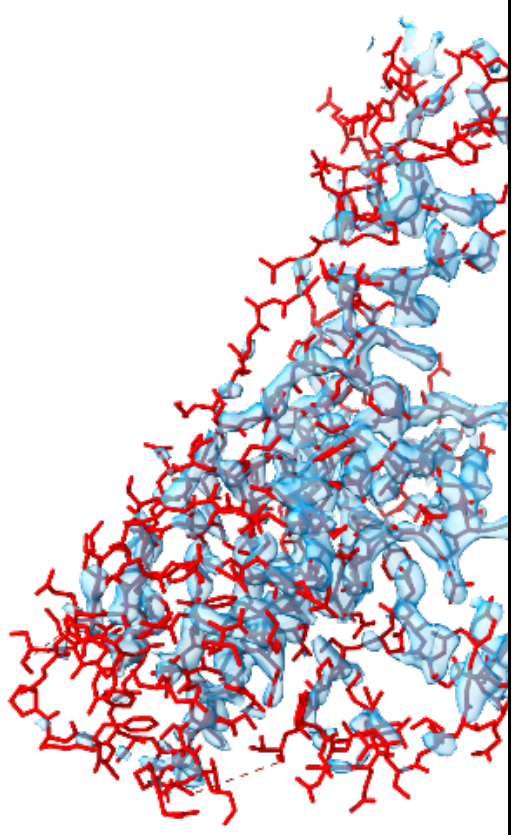	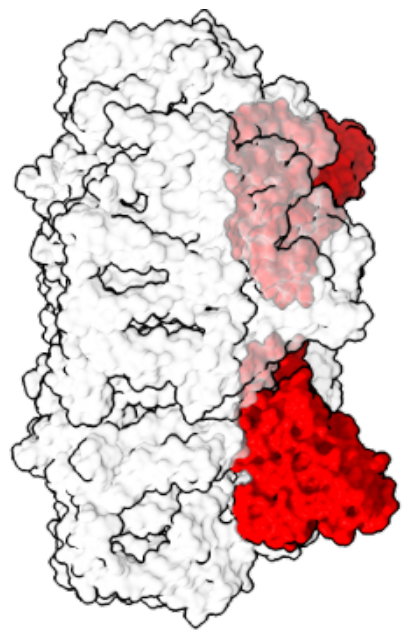
T,t	**Photosystem II reaction center protein Tc** (PsbTc)	3.82	>P61839<br>MEALVYTFLLVSTLGIIFFAIFFREPPKISTK<br>K	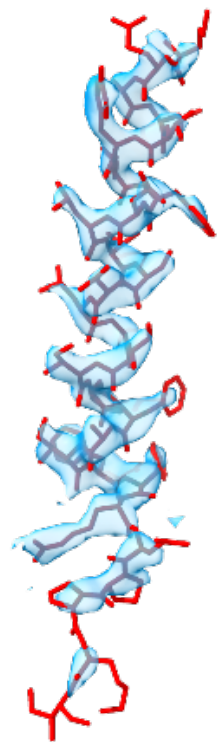	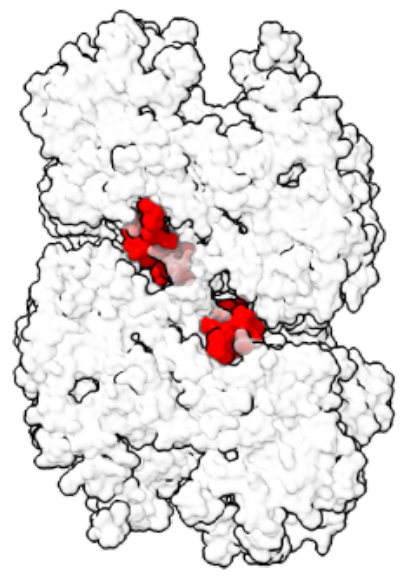
U,u	**Photosystem II reaction center protein Tn** (PsbTn)	3.17	>Q39195<br>EPKRGTEAAKKKYAQVCVTMPTAKICRY	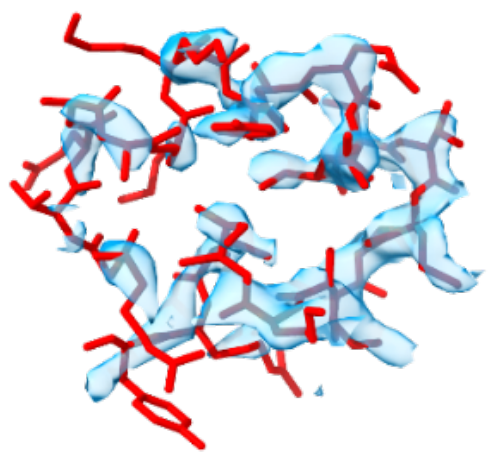	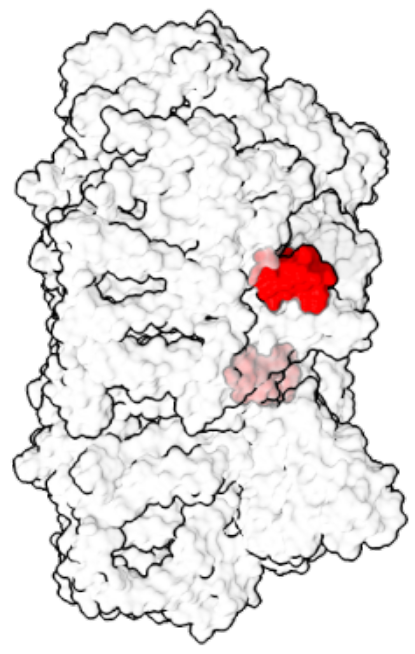
X,x	**Photosystem II reaction center protein X** (PSII-X)	4.18	>Q9SKI3<br>AGSGISPSLKNFLLSIASGGLVLTVIIGVVV<br>GVSNFDPVKRT	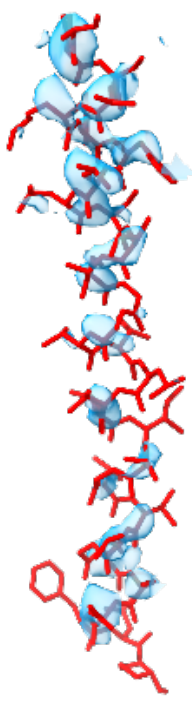	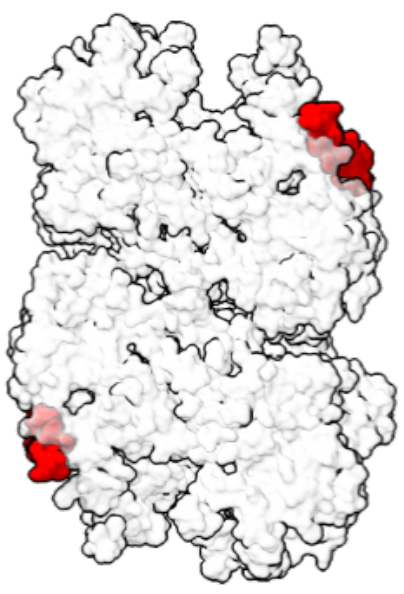

Supplemental Table 3: Details of various ligands found within the high-light structure.

Ligand Name	3-letter Code	# of ligands (full dimer)
β-CAROTENE	BCR	12
CHLOROPHYLL A	CLA	70
CHLORIDE ION	CL	4
DIGALACTOSYL DIACYL GLYCEROL (DGDG)	DGD	6
FE (II) ION	FE2	2
PROTOPORPHYRIN IX CONTAINING FE	HEM	2
WATER	HOH	52
1,2-DIPALMITOYL-PHOSPHATIDYL-GLYCEROLE	LHG	8
1,2-DISTEAROYL-MONOGALACTOSYL-DIGLYCERIDE	LMG	6
CA-MN4-O5 CLUSTER	OEX	2
PHEOPHYTIN A	PHO	4
2,3-DIMETHYL-5-(3,7,11,15,19,23,27,31,35-NONAMETHYL-2,6,10,14,18,22,26,30,34-HEXATRIACONTANONAENYL-2,5-CYCLOHEXADIENE-1,4-DIONE-2,3-DIMETHYL-5-SOLANESYL-1,4-BENZOQUINONE	PL9	2
1,2-DI-O-ACYL-3-O-[6-DEOXY-6-SULFO-ALPHA-D-GLUCOPYRANOSYL]-SN-GLYCEROL	SQD	2
